# Measuring empathy online and moral disengagement in cyberbullying

**DOI:** 10.3389/fpsyg.2023.1061482

**Published:** 2023-04-27

**Authors:** Sofia Mateus Francisco, Paula da Costa Ferreira, Ana Margarida Veiga Simão, Nádia Salgado Pereira

**Affiliations:** CICPSI, Faculdade de Psicologia, Universidade de Lisboa, Alameda da Universidade, Lisbon, Portugal

**Keywords:** assessing empathy online, measuring moral disengagement in cyberbullying, instruments, cyberbullying, adolescents

## Abstract

This investigation intends to explore how adolescents report empathy in online contexts and moral disengagement in cyberbullying incidents, and how these two constructs are related. To accomplish this goal, three studies were conducted considering the need to develop new instruments to uncover this new approach of measuring empathy and moral disengagement. In the first study, we adapted the Portuguese version of the Empathy Quotient-short form to online contexts, which resulted in the Empathy Quotient in Virtual Contexts (EQVC). We also developed the Process Moral Disengagement in Cyberbullying Inventory (PMDCI), in order to assess moral disengagement in these specific situations. In the second study we conducted exploratory factor analyses (*N* = 234) of these instruments. Finally, in the third study, we conducted confirmatory factor analyses (*N* = 345) of both instruments. These results showed how adolescents reported empathy in online contexts and moral disengagement in cyberbullying incidents. Specifically, empathy revealed a bi-dimensional structure including difficulty and self-efficacy in empathizing (Cronbach’s *α* = 0.44, 0.83, respectively), whereas process moral disengagement revealed four unidimensional questionnaires including locus of behavior, agency, outcome, and recipient (Cronbach’s *α* = 0.76, 0.65, 0.77, 0.69, respectively). Furthermore, a correlational analysis was also performed of both constructs, and we also considered the variable sex. Results showed that difficulty in empathizing was negatively associated with sex (with girls revealing more difficulty than boys) and all moral disengagement mechanisms except for behavior. Moral disengagement was positively correlated with sex, suggesting boys morally disengaged more from cyberbullying. The instruments provided new insights on how empathy and moral disengagement can be specific to online contexts and cyberbullying situations, and how they can be used in educational programs to promote empathy and gain insight on moral disengagement within this phenomenon.

## 1. Introduction

People are not only autonomous agents, but also function as the product of a reciprocal interplay of intrapersonal, behavioral, and environmental events (Bandura, 1986). Therefore, this investigation is based on the Social Cognitive Theory, which adopts an agentic perspective. Specifically, in this investigation we explore the relation between two intrapersonal factors that are proven to play an important role in cyberbullying involvement, which are empathy and moral disengagement.

Cyberbullying is a pervasive problem in our society, as it increases and causes harmful consequences in the lives of children and adolescents (Kowalski et al., 2014). Considering this, it is of utmost importance to be familiar with factors that play a role in preventing or reinforcing this type of behavior (Lo Cricchio et al., 2020). Many factors have been studied in relation to cyberbullying, such as empathy and Moral Disengagement (MD) (Marín-López et al., 2020; Ferreira et al., 2021).

When someone is involved in conflicts, empathy allows us to empathize with and understand others, but also helps us to predict the type of response of others (i.e., aggressive). Thus, it is assumed that empathy can serve as a control mechanism in conflict dynamics (Klimecki, 2019), which may include aggressive behavior (Tampke et al., 2020), such as in bullying and cyberbullying.

Therefore, empathy plays an important role in cyberbullying, however, it does not explain or predict it (Pfetsch, 2017). In fact, empathy has been found to be negatively related to cyberbullying perpetration (Garaigordobil, 2015). With respect to bystander behavior, empathy has been found to be an important factor for increasing prosocial behavior (Barlińska et al., 2018), therefore it can be considered a protective factor (Zhu et al., 2021).

Considering that cyberbullying may be seen as intentional and repeated acts of aggression toward peers (Hinduja and Patchin, 2009), involving moral aspects (Romera et al., 2021), it is also crucial to understand moral (dis) engagement within this phenomenon, which is an important risk factor in the cyberbullying cycle (Gao et al., 2020; Romera et al., 2021). With respect to bullying, Wang and Goldberg (2017) suggested that MD predicted and increased bullying perpetration in adolescence, and Thornberg et al. (2019) also found that bullying perpetration could also lead to MD. That is, MD impacted aggressive conduct, and aggressive conduct also impacted MD progressively over time (Bandura, 1999). For example, Falla et al. (2020) found that moral disengagement also had an impact on bullying victims, since cognitive restructuring (i.e., moral justification, euphemistic language and advantageous comparison) influenced the association between victimization and later, bullying behavior. Moreover, that same set of MD mechanisms were the single strongest predictor of both offline and online bullying (Romera et al., 2021). Thus, mechanisms of MD prevent individuals from feeling unpleasant emotions when perpetrating transgressions (Mazzone et al., 2019). Falla et al. (2021) argued that MD mechanisms may lead to a decrease in empathy, considering that the first seem to promote aggressive behavior, and the latter is related to prosocial behavior. Thus, considering that empathy seems to play an important role in moral development (Cameron et al., 2019), assessing both constructs with regards to online contexts and understanding the possible relation between them, may provide an important contribution to the field. For example, Francisco (2022) discussed that empathy can be viewed as a shield for the impulsive use of MD mechanisms, since they found that when adolescents did not spontaneously use MD mechanisms to justify aggressors’ and/or bystanders’ cyberbullying behavior, they tended to show empathic responses instead. Moreover, Haddock and Jimerson (2017) studied the correlation between MD and empathy and found that this correlation was statistically significant and negative. Specifically, these authors found that affective empathy and cognitive empathy both significantly predicted MD. Accordingly, as MD increased, affective and cognitive empathy decreased. In general, students who had higher scores in MD, tended to have lower scores in empathy. Despite the differences that can occur in feeling empathy online and the activation of MD mechanisms with respect to cyberbullying incidents, we believe that a similar relationship might occur between these constructs, since it occurs within bullying (Haddock and Jimerson, 2017). Therefore, this study aims to assess adolescents’ perceived empathy with regards to online contexts and their MD in cyberbullying situations with two new instruments. We also proposed to understand the relationship between the two constructs, considering adolescents’ perspectives, because the MD instrument was developed according to adolescents’ point of view regarding cyberbullying scenarios.

### 1.1. Measuring adolescents’ perceived empathy regarding online contexts

#### 1.1.1. The importance of the online context

This study is positioned within the perspective of empathy online, namely that it is possible to express “traditional empathic characteristics such as concern and caring for others … through computer-mediated communications” (Terry and Cain, 2016, p. 1). In fact, this study focuses specifically on empathy in virtual contexts, because empathy itself is not online, but rather, occurs within individuals as they establish interpersonal relations in virtual contexts. To date, few studies have considered this specificity and have assessed empathy with adapted instruments. That is, few studies have considered the online characteristics of empathy, when studying cyberbullying. Nonetheless, some studies have already taken empathy in virtual contexts into account. For example, Carrier et al. (2015) and Manasia and Chicioreanu (2017) found that virtual empathy was positively related with empathy in face-to-face interactions, however, virtual empathy was lower for both sexes. Complementarily, Marín-López et al. (2020) found no differences between the different cyberbullying roles with respect to online empathy. Considering the scarce literature with respect to empathy in virtual contexts and cyberbullying (Marín-López et al., 2019, 2020), it is crucial to develop further research in this area of knowledge.

Assessing empathy is important to explain bystanders’ role in cyberbullying situations. For instance, Macaula and Boulton (2017) found that when comparing positive bystanders’ responses in bullying and cyberbullying, the rate of responses tended to be higher in cyberbullying. Moreover, this type of responses in both bullying and cyberbullying was positively and moderately correlated with empathy. Also, positive bystander responses tended to increase, as a result of cyberbullying severity. Another study (Schultze-Krumbholz et al., 2018) found that higher levels of both cognitive and affective empathy were associated with prosocial defending, when compared to passive bystander behavior. Notwithstanding, the research presented above considered measures of empathy without accounting for the online context.

From a phenomenological perspective, Fuchs (2014) proposed that it is not possible for empathy to occur in online contexts, since we lose our perceptual access to other individuals’ physical presence, and thus, we lose our direct empathic access to others. Accordingly, for empathy to occur, we need to perceive other individuals’ “lived body” (see Osler, 2021), and this is not possible in online “disembodied communication” (Fuchs, 2014, p. 167). Moreover, the temporal delay and the loss of perceptual queues (i.e., the perception we have is not apprehended by all our sensory capabilities) that occurs in technological mediated communication prevents us from perceiving someone’s physical and emotional experience. This was not a concern in face-to-face interactions, but do come into play in online interactions (Osler, 2021). Despite these perspectives, we believe it is possible to feel empathy in online contexts, even if individuals do not see others in person. We consider this to be true because empathic skills can be developed through the use of virtual reality (e.g., Bertrand et al., 2018), which is also different from face-to-face interactions. Moreover, although there are differences between online and offline communication, individuals tend to use other cue systems at their disposal, with the objective of promoting and detecting these cues, as well as developing relationships (Walther, 1995). Therefore, if relationships can be developed, empathy can also be possible in online interactions. In fact, through interpersonal communication online, individuals are able to infer what others might be thinking/feeling in a certain situation (Carrier et al., 2015). Nonetheless, the specificities of online contexts, may make it difficult for empathic reactions (Terry and Cain, 2016). Despite the fact that few studies have investigated empathy in virtual contexts and its specificities, it has been already proven that empathy can be experienced online. For example, Preece (1999) found that empathy online was quite common in support groups, which corroborates our position. This author discussed that the difference between synchronous and asynchronous systems impacts communication. Firstly, the pace of interaction is very different between these systems, that is, in one it is almost immediate, whereas in the other, it can take much more time (i.e., hours, days, or weeks differing from the platform). Moreover, another important difference is regarding the mode of expression, and other features that allowed nonverbal expression, whereas in the asynchronous system the primary mode is written text. It is important to highlight that this investigation is from the 1990’s, and several features of online communication have changed. However, more recent studies have found that text-type emoticons and graphic emojis are processed in a similar way to in-person facial expressions (Gantiva et al., 2019), and participants who viewed text-type emoticons exhibited face imitation mirroring (O’Neil, 2013). Therefore, we can argue that it is possible to feel empathy when interacting in virtual contexts.

#### 1.1.2. Gaps in existing scale development

Considering the importance of accounting for online features in measuring empathy, we sought new instruments on empathy that were developed according to the online context. To date, we found three instruments directly adapted from the Basic Empathy Scale (Jolliffe and Farrington, 2006), that is, the Virtual Empathy Scale (Carrier et al., 2015), the Online Empathy Questionnaire (Marín-López et al., 2019) and the Virtual Basic Empathy Scale (Manasia and Chicioreanu, 2017). Also, another instrument was adapted by García-Pérez et al. (2016) based on the Basque version (Gorostiaga et al., 2014) of the *Test de Empatía Cognitiva y Afectiva* (TECA) from López-Pérez et al. (2008). Additionally, Happ and Pfetsch (2015) developed the Media-Based Empathy (MBE) Scale (original name *Skalazumedienbasierter Empathie*) based on a pool of items according to the Interpersonal Reactivity Index (Davis, 1980) and an instrument to assess media empathy by Früh and Wünsch (2009), which included media concern, affective media empathy, cognitive media empathy, and immersion in video games, with items related to different types of media, as well as fictional and real people. Of all these instruments, only the Online Empathy Questionnaire (Marín-López et al., 2019) was used in relation to cyberbullying behavior.

Despite the valuable contributions in terms of the aforementioned instrument development and validity studies, and after a detailed analysis of the respective items, we found that the Empathy Quotient (EQ) by Baron-Cohen and Wheelwright (2004) would be appropriate to reach our objectives. Specifically, these authors defined empathy as “The drive or ability to attribute mental states to another person/animal and entails an appropriate affective response in the observer to the other person’s mental state” (Baron-Cohen and Wheelwright, 2004, p. 168). The term “quocient” derives from the Latin word “quotiens” which means “how much” or “how many” (Baron-Cohen and Wheelwright, 2004, p. 166). According to this perspective (Baron-Cohen, 2011), that if individuals only focus on their own problems or interests, they are likely to feel less empathy. In fact, when individuals feel empathy, they are able to identify what others are thinking or feeling and are able to provide an adaptive emotional response. Thus, this view of empathy entails two fundamental stages: recognition and response. Accordingly, empathy occurs when there is recognition and an adaptive response, which helps avoid hurting others and fosters prosociality.

Some studies have provided evidence that the Empathy Quotient was the third most used instrument (e.g., Ilgunaite et al., 2017) and a recent a meta-analysis by Hall and Schwartz (2019) determined that it was the second most used instrument in research. For this investigation the aim was to choose an instrument that had been widely used and already validated for several countries (e.g., Redondo and Herrero-Fernández, 2018), but that also included items assessing accurate interpersonal perception (Hall and Schwartz, 2019), since it is an important feature when assessing empathy, specifically in the virtual contexts, as is the case with this study. Moreover, we preferred to adapt the short form of this questionnaire, which had already been developed by Wakabayashi et al. (2006), and adapted for the Portuguese population (Rodrigues et al., 2011). Our study provides an important contribution, since it proposes to adapt this last version of the instrument to a younger population and for online contexts.

#### 1.1.3. Goals of the present work

Considering the literature reviewed, one of the main purposes of this study is to present and evaluate a new version of the Portuguese short form of the EQ for adolescents communicating online, entitled Empathy Quotient in Virtual Contexts (EQVC).

According to some of the literature, empathy can be developed over time (Gerdes et al., 2010) and may be considered a capacity (or ability), suggesting that individuals have the potential to empathize or not (Hall and Schwartz, 2019). In fact, in some circumstances, feeling empathy requires effort and cognitive costs, and therefore, individuals may avoid feeling empathy (Cameron et al., 2019). Thus, considering the specificities of the online environment and its consequences in interpersonal relationships, we felt the need to assess empathy that occurs specifically in virtual contexts. Moreover, empathy can be situation and context specific (Cameron et al., 2019) such as in cyberbullying situations. Nonetheless, despite the widespread consensus that empathy is predetermined by circumstances (Barlińska et al., 2013), none of the empathy definitions clearly state that empathy can decrease in some situations. That is, for example, in a bullying situation, an individual might feel empathy, however, if a similar situation occurs online, the same individual might not feel the same degree of empathy. This is one of the reasons we opted to adapt an empathy instrument for online contexts, as it may be more difficult for individuals to feel empathy toward others in these digital environments (Pfetsch, 2017).

### 1.2. Assessing moral disengagement in cyberbullying situations

According to the Social Information Processing theory (Walther, 2015), the lack of nonverbal cues in many forms of computer-mediated communication (CMC) causes relational information to be exchanged more slowly. As a result, relationships develop more slowly *via* CMC than in face-to-face interactions, but eventually reaches equivalent levels of development (Walther, 1992). Moreover, the scarcity of social–emotional cues and the easiness of sharing media content may facilitate the use of certain MD mechanisms (Runions and Bak, 2015).

Before cyberbullying had been linked to MD (for a meta-analytic review see Zhao and Yu, 2021), Suler (2004) had already investigated some characteristics of the online world that impacted individuals’ online actions. For instance, Suler (2004) argued that in cyberspace, people tended to say and do things that normally they would not in face-to-face interactions. Suler explained how dissociative anonymity, invisibility and asynchronicity facilitated online disinhibition. He also discussed other factors, however considering cyberbullying situations, those three seemed more important. Specifically, Suler defended that dissociative anonymity allowed people to distance themselves from their online behavior, which is one of the main principles that helps explain online disinhibition. Furthermore, the fact that it was possible to be invisible in online interactions also amplified the disinhibition effect because people did not worry about how they looked when they communicated online (Suler, 2004). Thus, considering that the virtual online world seems to be characterized by a degree of disinhibition (Suler, 2004), which is a crucial social environment for MD (Bandura et al., 1996), cyberbullying behavior will be more frequent for individuals with higher MD (Zhao and Yu, 2021). That is, the lack of emotional cues in online settings may result in dehumanization (i.e., depriving another person from human qualities; Bandura, 2002), whereas the ease with which young people share information online, may facilitate the diffusion and displacement of responsibility (distributing the responsibility for several individuals or attributing the responsibility to an authority; Bandura, 2002). Accordingly, ambiguous communication, which is common online, may provoke cyber aggression which is justified by the perceived blame of the other (Runions and Bak, 2015). Moreover, the same authors argued that young people are technologically more immersed, and media attention is increasing regarding extreme cases of cyberbullying. Hence, the relationship between online contexts and the use of MD mechanisms stresses the importance of assessing the construct in terms of specific behavior that occurs online, which in the case of this study, is cyberbullying behavior.

To our knowledge, few studies have accounted for MD in online settings. For instance, Paciello et al. (2020) found that online MD and offline MD were correlated, even though they were distinct constructs. Moreover, they found that depending on the degree of externalizing behavior, the importance of online and offline MD was different. Specifically, cyberbullying was only significantly related to online MD for low externalizing adolescents, whereas for medium externalizing behaviors, both online and offline MD were significant. For high externalizing participants, only offline MD was significant. Complementarily, Marín-López et al. (2020) found that online MD was generally higher for children who were involved in cyberbullying (specifically cyberbullies and cybervictims), when compared to those who were not.

Some instruments have already been developed to assess MD in cyberbullying context. One of the first measures of MD in cyberbullying situations was from Bussey et al. (2015), in which they reworded 8 items from the MD scale by Bandura et al. (1996). Later, Day and Lazuras (2016) developed the Cyberbullying-specific Moral Disengagement Questionnaire (CBMDQ-15) which is a 15-item scale based on thematic analysis of focus group interviews with undergraduate students, from where eight themes reflecting the MD mechanisms (Bandura, 1991) emerged. In recent years, two more questionnaires were developed. Marín-López et al. (2019) developed the Moral Disengagement through Technology Questionnaire, also based on Bandura et al. (1996) and adapted to online interactions. Additionally, Cuadrado-Gordillo and Fernández-Antelo (2019) combined two different questionnaires (Day and Lazuras, 2016; Meter and Bauman, 2018) and transform the different types of aggression to online contexts. More recently, Paciello et al. (2020) developed the Online Moral Disengagement scale referring to “online social settings and misbehavior” (Paciello et al., 2020, p. 191).

Despite the aforementioned instruments to assess MD in online interactions (e.g., Paciello et al., 2020) and cyberbullying situations (e.g., Bussey et al., 2015), we consider that the development of a new instrument would be beneficial to assess the construct as a process for the Portuguese population, rather than just an adaptation to the Portuguese language. The main objective was to develop an instrument that could capture adolescents’ view regarding cyberbullying phenomenon, and MD as a process. That is, we intended to follow Bandura’s (2002) Social Cognitive Theory, but we also aimed to complement this perspective with new information that participants may report regarding MD in cyberbullying situations. We consider this important because most instruments presented were only adaptations to online contexts, without considering adolescents’ view of the phenomenon. Thus, this study also aims to present the new developed instrument to assess MD regarding cyberbullying situations (Process Moral Disengagement in Cyberbullying Inventory [PMDCI]), as well as to evaluate its psychometric properties.

### 1.3. Adolescents’ perceived empathy online and moral disengagement in cyberbullying

Empathy is central for moral development (Cameron et al., 2019), as it can be an antecedent of moral attitudes (Hyde et al., 2010). Additionally, as empathy can be considered the base for more abstract moral concepts, as well as attitudes toward society, it is probably an antecedent of subsequent moral attitudes, such as MD. For example, Hyde et al. (2010) postulated that both MD and empathy share an element of disengagement, that is, MD is directed at society and its values, whereas empathy can be considered more person-specific. For instance, moral self-censure derives from how aggressors regard the individuals they harm, therefore, if they perceive another person as human this can activate empathic reactions through perceived similarity (Bandura, 1992). Moreover, Francisco (2022) found that when spontaneously talking about fictitious cyberbullying scenarios, participants who tended to use less MD mechanisms to justify aggressors’ and bystanders’ cyberbullying behavior, showed more empathic responses. Thus, empathy and MD seem to be related, as they can be seen as opposite sides of the same coin, and therefore, highlighting the importance of a concerted work including empathy and MD, with the aim of increasing prosocial behavior online (Francisco, 2022). Moreover, MD and empathy are two relevant personal factors in cyberbullying bystanders’ behavior. However, the relationship between the two constructs is not fully understood (Marín-López et al., 2020). Thus, taking this into account, and considering the virtual world and cyberbullying involvement, we propose that adolescents’ perceived empathy regarding online contexts may be related to MD with cyberbullying situations.

It is known that gender can have an impact on several individual factors, such as empathy and MD. For example, Falla et al. (2021) found gender differences with respect to empathy and MD in relation to bullying. Specifically, the authors found that girls had higher scores on both cognitive and affective empathy, and that boys had higher scores on several MD mechanisms, such as cognitive restructuring, minimizing responsibility, distorting consequences and dehumanizing. Thus, considering these gender differences we argue whether gender can have an impact on the variables of this study. Therefore, we question: (1) Is there a relationship between Empathy in virtual contexts and MD related to cyberbullying situations? If so, how are these constructs related?; and (2) What is the role of gender in empathy in virtual contexts and MD in cyberbullying situations?

In order to reach our objectives and answer our research questions, we present three distinct studies. A first study explores the initial adaptation of the EQVC and the preliminary development of the PMDCI. A second study presents the exploratory psychometric evidence of the EQVC and the PMDCI, whereas a third study shows the confirmatory analyses of the instruments and a correlational study of the two constructs.

## 2. Study 1- Adaptation of the EQVC and preliminary development of the PMDCI

### 2.1. Method

#### 2.1.1. Ethical aspects

For all the studies presented, authorization to complete the questionnaires in the online context was granted by the Ministry of Education of Portugal, the Portuguese National Commission of Data Protection, the Deontology Committee of the researchers’ institution, the schools’ boards of directors, the teachers, the parents and the adolescents themselves. Before the completion of the questionnaires, students were informed that psychological assistance was available if needed, considering the sensitivity of the subject in study. Additionally, students were informed that all information collected was anonymous and confidential and that they could quit at any time if they were not comfortable. This study was not preregistered. Further information regarding the initial adaptation and construction of the instruments, all items (Portuguese version), and additional information are available in the Supplementary material.

### 2.2. Initial adaptation of the EQVC

All the 22 items from the Portuguese version of the EQ short form were converted to the online context considering its specificities. Later, these items were compared to the original version in English, by a bilingual Portuguese-English teacher. Considering the different populations from the original version (i.e., adults) and ours, some modifications were made to simplify the items and make them more comprehensible for the adolescent population. Lastly, small changes were made considering students’ feedback in the face validity session (see Supplementary Appendix A.1 and Supplementary Appendix Table A.1).

### 2.3. Initial construction of the PMDCI

#### 2.3.1. Participants

Thirty-four 9th grade students (Mage = 14.29, SD = 0.72, 53% female) participated in an in-depth semi-structured interview with fictitious scenarios.

#### 2.3.2. Procedure

**Figure 1:**
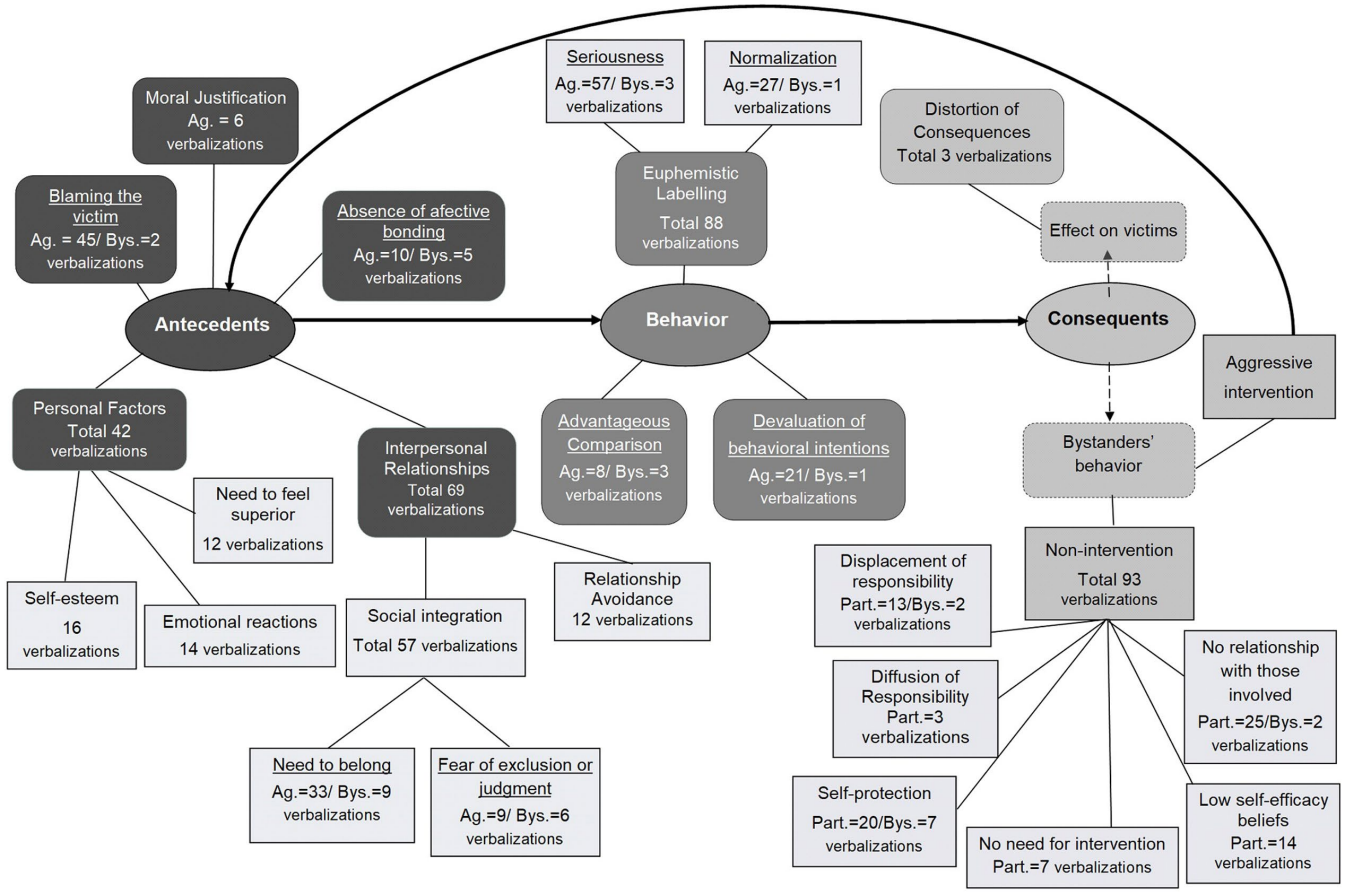
Procedural model of cyberbullying in the perspective of participants, as bystanders of the scenarios. Ag., aggressors’ behavior; Bys., bystanders’ behavior; Part., participants’ bystanders behavior in the scenarios. From Francisco et al. (2022).

A qualitative study was conducted to explore adolescents’ MD in cyberbullying situations. In-depth semi-structured interviews with scenarios were conducted and *verbatim* transcribed. Later, we performed a content analysis with a mixed approach (deductive/inductive), based on the Social Cognitive Theory (Bandura, 2002). The coding units we established were adolescents’ written verbalizations with meaning (Amado et al., 2014), summing a total of 396 verbalizations, which were analyzed. We performed an initial phase, where categories were created, and a re-checking phase, where a set of verbalizations were analyzed by two other researchers and adjustments were made to the operational definition of the categories. Finally, two independent coders rated the data. Inter-rater reliability was excellent, as mentioned in the literature (McGraw and Wong, 1996), with an ICC = 0.99, with a 95% confident interval = 0.99–0.99. From this analysis, the categorization process went beyond the Social Cognitive Theory. That is, several categories of MD mechanisms emerged from the analysis, as well as other attributions (Figure 1), both regarding aggressors’ and bystanders’ behavior from the scenarios (see Francisco et al., 2022 for a detailed description).

It is important to highlight that we considered MD as a process, since several mechanisms tend to be used before the aggression, during the behavior and after as consequents of the behavior, as presented in Figure 1. Thus, considering this novel approach, the qualitative data was the starting point of the development of the PMDCI because we sought to develop an instrument that could capture adolescents’ beliefs and perspective of this phenomenon as accurately as possible. Hence, from the categories that emerged from the content analysis, we created the items for the PMDCI. All the procedures regarding scale development can be found in the Supplementary Appendix A.2.

### 2.4. Results

Study 1 allowed us to develop the EQVC and the PMDCI. The EQVC is composed of 22 items in Portuguese, for the adolescent population. The final items were translated into English, for the purpose of presenting this investigation (Supplementary Appendix Table A.8). As for the PMDCI, it was an instrument about the psychological mechanisms adolescents use to justify their cyberbullying-related actions, in the perspective of possible aggressors and bystanders (Supplementary Appendix Tables A.2–A.6). The inventory begins with a brief introduction about adolescents’ daily use of ICT. The PMDCI (Supplementary Appendix A.3) is also composed of two scales (the aggressor’s and bystander’s perspective), because when speaking freely about the cyberbullying scenarios, adolescents tended to use MD mechanisms to not only legitimize cyberbullies’ actions, but also to approve cyber bystanders’ aggressive behavior. The PMDCI also includes a Non-Intervention scale. However, for the purpose of this work, only the bystander scale was used, since it is part of a larger investigation that aims to improve bystanders’ prosocial behavior online. The Bystander Scale of the PMDCI is composed of 36 items (24 regarding MD mechanisms, 3 regarding the devaluation of behavioral intention, and 9 items in the attribution category). All items were presented with a Likert scale from 1 (*totally disagree*) to 4 (*totally agree*).

## 3. Study 2 – Preliminary testing and exploratory psychometric evidence of the EQVC and 363 the PMDCI

### 3.1. Method

#### 3.1.1. Participants

A total of 234 students participated in the exploratory factor analysis (EFA) study (*M_age_* = 13.24; *SD* = 1.18; 51.7% girls), 35.9% of whom were in the 7th grade, 25.6% were in the 8th grade and 38.5% were in the 9th grade (Supplementary Appendix A.4). All 234 participated in the EFA of the EQVC and 230 participated in the EFA of the PMDCI.

#### 3.1.2. Procedures

The new created version of EQ (EQVC) and the new developed instrument (PMDCI) were administered on-line in a classroom context, individually with the guidance of an educational psychologist. Students took approximately 40 minutes to complete both questionnaires. After the data gathering, EFA was conducted with FACTOR 10.10.02 (Ferrando and Lorenzo-Seva, 2017) to understand the factorial structure of both instruments. Specifically, we intended to explore if the EQVC yielded the same structure of the EQ-short form (Portuguese version), or if considering the new context and different population, the structure of the instrument would change. Regarding the PMDCI, since it was developed considering the four loci (i.e., Behavior, Agency, Outcome and Recipient) and the respective MD mechanisms, we intended to evaluate the best way to validate the instrument. That is, we were interested in understanding if the instrument should be considered as a single scale, or if it should be regarded as a questionnaire with different scales (i.e., one scale for each locus) involving the distinct locus of the MD.

### 3.2. Results

#### 3.2.1. Exploratory evidence of the EQVC

In order to uncover the underlying structure of the EQVC, we performed an EFA (see Supplementary Appendix A.5 for more details). We present the correlations and descriptive statistics of all items, including skewness and kurtosis (Supplementary Appendix Table A.7). Regarding univariate normality, all variables were approximately normally distributed according to the literature, with skewness absolute values less than 2 and kurtosis absolute values less than 2 (George and Mallery, 2016). We also analyzed multivariate normality accordingly to Bollen and Long (1993), where multivariate normality is accepted if Mardia’s coefficient is lower than P (P + 2), considering P the number of observed variables. Considering that the EQVC presented 22 observed variables, Mardia’s coefficient for skewness of 78.41 < 22(22 + 2) = 528 and for kurtosis is 605.06 > 22(22 + 2) = 528. Moreover, as for the correlation matrix, we used polychoric correlations (Muthén and Kaplan, 1985; Brown, 2006) (Supplementary Appendix Table A.7). Furthermore, before proceeding to the EFA results, Kaiser–Meyer–Olkin (KMO) and Bartlett Sphericity were assessed. As for KMO it was 0.89 revealing sampling adequacy, and Bartlett Sphericity test was χ^2^(_231_) = 2543.4 (*p* < 0.001), which indicated that we could proceed with factor analysis. In order to retain the appropriate number of factors we used Horn Parallel analyses (O’Connor, 2000). In the FACTOR program (Ferrando and Lorenzo-Seva, 2017) the Optimal Implementation of Parallel Analysis (Timmerman and Lorenzo-Seva, 2011) suggested that two factors should be extracted. We used the Unweighted Least Squares (ULS) method for factor extraction. Specifically, Robust Factor Analysis based on the Robust Unweighted Least Squares (RULS) was used to fit the factor solution. Robust Promin Rotation was used to achieve factor simplicity (Lorenzo-Seva and Ferrando, 2019). As according to the literature (Bandalos and Finney, 2010), we took into account all items with structure coefficients superior to 0.30, and no items revealed loadings greater than 0.40 on the two factors (Supplementary Appendix Table A.2). According to the literature (McDonald, 1999), goodness-of-fit values (*GFI* = 0.98) and (*AGFI* = 0.98), residuals statistics (*RMSR* = 0.06) were good. The EQVC presented 48% of the explained variance. We then compared the bi-factorial model to the unifactorial model (Supplementary Appendix A.6 and Supplementary Appendix Table A.9). Considering the results, we decided to keep the bi-factorial model since the percentage of explained variance was higher. Regarding reliability, McDonald’s Omega (Hayes and Coutts, 2020) was also assessed for both factors: factor 1 presented ω = 0.68, 95% CI [0.58, 0.74], showing acceptable reliability, and factor 2 presented ω = 0.91, 95% CI [0.88, 0.93], with excellent reliability (Supplementary Appendix A.6).

Later, we conducted a Multidimensional Normal-ogive Graded Response Model (Reckase, 1985), whose parameters can be seen in Supplementary Appendix Table A.8, as well as the item loadings. This model presents a discrimination parameter (*a*), which is important in the preliminary adjustment of questionnaires and item selection (Matteucci and Stracqualursi, 2006). Most items revealed moderate item discrimination, however, items 1, 4, and 5 revealed low item discrimination, presenting values between 0.424 and 0.586, as indicated in the literature (Baker, 2001). Item discrimination reveals how well an item differentiates individuals scoring high and low on the latent ability being measured (Depaoli et al., 2018). Then, we performed the analysis again without items 1, 4, and 5 to see how the model change. Lastly, we had some participants with Weighted Mean-Squared Index larger than 2.0 (Ferrando et al., 2016), thus, these participants were removed and the analysis was performed again. Table 1 shows a comparison between 4 proposed EFA models: (1) with all participants and all items, (2) with all participants and without items 1, 4 and 5, (3) without infit/outfit participants and all items and (4) without infit/outfit participants and without items 1, 4, and 5.

**Table 1:** Proposed bi-factorial model parameters of the EQVC.

	Model 1		Model 2		Model 3		Model 4	
Mardia’s coefficient skewness	78.41 < 22(22 + 2) = 528		54.56 < 19(19 + 2) = 329		78.81 < 22(22 + 2) = 528		52.78 < 19(19 + 2) = 329	
Mardia’s coefficient kurtosis	605.06 > 22(22 + 2) = 528		465.91 > 19(19 + 2) = 440		588.02 > 22(22 + 2) = 528		449.76 > 19(19 + 2) = 440	
Kaiser–Meyer–Olkin	0.89		0.90		0.91		0.92	
Bartlett sphericity	χ^2^_231_ = 2543.4 (*p* < 0.001)		χ^2^_171_ = 2292.2 (*p* < 0.001)		χ^2^_231_ = 2415.8 (*p* < 0.001)		χ^2^_171_ = 2356.1 (*p* < 0.001)	
% Explained variance	48%		52%		51%		55%	
GFI	0.98		0.99		0.99		0.99	
CFI	1.00		1.00		1.00		1.00	
RMSR	0.057		0.055		0.051		0.049	
RMSEA	0.028		0.032		0.015		0.018	
α	0.68	0.91	0.64	0.91	0.68	0.92	0.64	0.92
ω (95%)	0.68 [0.58, 0.74]	0.91 [0.88, 0.93]	0.64 [0.54, 0.72]	0.91 [0.88, 0.93]	0.68 [0.57, 0.74]	0.92 [0.90, 0.94]	0.63 [0.53, 0.72]	0.92 [0.90, 0.94]

α and ω were calculated for difficulty in empathizing and self-efficacy regarding empathy, in all models. ω is assessed with 95% confidence interval.

The elimination of participants improved the % of explained variance (from 48 to 51%); the RMSEA and the RMSR were the fit indices that had better improvement. Moreover, the elimination of the 3 items improved the model essentially in terms of % explained variance (from 48 to 55%), and also the same indices as described above. Considering these improvements, we conducted Confirmatory Factor Analysis (CFA) with this structure.

#### 3.2.2. Exploratory factor analysis of the PMDCI

With the aim of assessing the structure of the PMDCI, we performed an EFA with data from 230 participants to the 5 scales included in the questionnaire (4 scales regarding Loci of MD and 1 scale regarding Attributions for the cyberbullying behavior), considering the Bystanders’ perspective (i.e., Bystander scale). We present the correlations and descriptive statistics of all items, including skewness and kurtosis (Supplementary Appendix Table A.10).

Regarding univariate normality, most of the variables were normally distributed, with skewness absolute values less than 2 (Bollen and Long, 1993), with the exception of the items from the Attribution Scale. Regarding kurtosis, all variables had less than 5 in absolute value. With respect to multivariate normality, according to Bollen and Long (1993), it is accepted if Mardia’s coefficient is lower than P(P + 2), considering P the number of observed variables. Moreover, as for the correlation matrix, we used polychoric correlations (Muthén and Kaplan, 1985; Brown, 2006). Furthermore, before proceeding to the EFA, Kaiser–Meyer–Olkin (KMO) and Bartlett Sphericity were assessed (Supplementary Appendix Table A.11). All scales had high KMO which revealed sampling adequacy, as well as a significant Bartlett Sphericity test, which indicates that we could proceed with factor analysis.

In order to retain the appropriate number of factors, we followed the same procedures used for the EQVC. Our EFA suggested that a single factor should be extracted of each scale of the PMDCI. As for the factor structure (Supplementary Appendix Table A.12), we took into account all items with structure coefficients superior than 0.30 (Bandalos and Finney, 2010). Regarding reliability, all scales reveal good internal consistency values (Supplementary Appendix Table A.11).

Regarding Explained Variance, all scales were above the minimum range, as according to the literature (Hair et al., 2014). As for the model fit indices, all scales presented satisfactory values of goodness-of-fit values and residuals statistics (Supplementary Appendix Table A.11), according to the literature (McDonald, 1999).

Later, we conducted a Multidimensional Normal-ogive Graded Response Model for unifactorial models (Samejima, 1969), whose parameters can be seen in Supplementary Appendix Table A.12, as well as the item loadings, for all the 5 scales. Considering the discrimination parameter values, it was concluded that all items from all scales revealed good discrimination (Baker, 2001), indicating that there was no need to remove items. Thus, we conducted CFA with the original structure of all 5 scales.

## 4. Study 3 – The confirmatory analyses of the instruments and a correlational study of the studied constructs

### 4.1. Method

#### 4.1.1. Participants

For the CFA, our sample consisted of 345 students (*M*age = 13.13; *SD* = 1.27; 51% boys), 40.5% of whom were in the 7th grade, 27.1% in the 8th grade and 32.4% in the 9th grade. Most students were Portuguese (85.8%). All 345 participated in the CFA of the EQVC and 342 participated in the CFA of the PMDCI, as well as in the correlational study.

#### 4.1.2. Procedures

Before proceeding to the CFA, univariate and multivariate normality of all scales were evaluated and the distributions were considered non-normal. This is consistent with the literature (Yuan and Bentler, 1998), since non-normality is prevalent in real data (Blanca et al., 2013) and it would dictate the possibilities in the data analysis, because structural equation modeling assumes the normality of latent variables (Bollen, 1989). Thus, several estimation methods were investigated and analyzed considering the nature of our data (for a detailed description see Supplementary Appendix A.7).

With this in mind, we attempted to analyze several estimation methods that could be applied to our data. As a way of summarizing our results, we only mentioned the ULS parameters in the text, as advised by Bollen (1989) because it does not make distributional assumptions regarding the observed variables. Moreover, the other estimation procedures are presented in the Supplementary Material and referred to when they are considered relevant.

For the CFA of the EQVC and PMDCI we used IBM, SPSS AMOS 24.0 (Arbuckle, 2019) and the *lavaan* package (Rosseel, 2012) in R Project (R Core Team, 2020). ULS and ML with Bollen-Stine Bootstrapping were conducted in AMOS, and ML with Satorra-Bentler correction and WLSMV were conducted using the *lavaan* package in R software. Several Fit Indices will be presented according to the different estimation methods (Supplementary Appendix A.7), and organized by their main classification. Considering that the covariance matrix might not be as asymptotically distributed as chi-square with the ULS method (Bollen, 1989), several statistics are not reported, such as the chi-square test and other fit indexes based on this statistic. Instead, we used the following fit indexes to ascertain the tested models: GFI, AGFI and PGFI (more information regarding Fit Indices are in the Supplementary Appendix A.9).

As for the correlational study, Spearman correlation coefficients were used to examine the relationship between the variables.

### 4.2. Results

#### 4.2.1. Confirmatory factor analysis of the EQVC

We examined the multivariate normality and considering that the critical ratio for both skewness and kurtosis was outside the interval of [−1.96, +1.96] (Byrne, 2010), some procedures were made to account for the non-normal distribution of the data. Thus, first several multivariate outliers were removed, and multivariate normality was assessed again. However, the distribution was still non-normal.

We tested various possible models so as to confirm the initial structure of the EQVC suggested by the EFA with confirmatory factor analysis. We attempted to test a model with all participants and no covariances (model 1), a model without outliers and no covariances (model 2) and a model without outliers and with covariances (Supplementary Appendix Table A.13 and Supplementary Appendix Figure A.1) between the error terms (model 3). From the results presented, we chose model 3, which according to the literature (Jöreskog and Sörbom, 1984; Cole, 1987; Blunch, 2008) presented good reference values [χ^2^(149) = 151.626, χ^2^/*df* = 0.793, GFI = 0.969, AGFI = 0.961, SRMR = 0.054, NFI = 0.930, PGFI = 0.759, PNFI = 0.810].

Despite the good fit of the model, several relationships between each factor and corresponding items were lower than the cut-off value of 0.5, as suggested in the literature (e.g., Bandalos and Finney, 2010). All unstandardized path coefficients^1^ were significant at *p* < 0.05, with the exception of item 3, which was equal to 0.05 (Supplementary Appendix Figure A.1). Moreover, the construct reliability scores were low for the Difficulties in Empathizing and higher than 0.80 (Hair et al., 2014) for the Self-efficacy regarding Empathy (Table 2). Thus, the second factor presented good construct reliability; however, the first, which only has 4 items, revealed low reliability. Convergent validity was low for both factors since the Average Variance Extracted (AVE) scores were lower than 0.50 (Henseler et al., 2009). Nonetheless, the Average Shared Variance scores below the AVE scores (Hair et al., 2014) indicated good discriminant validity of both factors. Additionally, the simplified model also presented lower Modified Expected Cross-Validation Index (MECVI), indicating that it has better validity in the population we are studying (Marôco, 2014).

**Table 2:** Validity measures of Model 3 from the EQVC.

Factors	Cronbach’s alpha	McDonald’s omega	CR	AVE	ASV	MSV
Difficulties in empathizing	0.44	0.45 [0.30–0.54]	0.39	0.18	0.12	0.12
Self-efficacy beliefs regarding empathy	0.83	0.83 [0.79,0.86]	0.83	0.26	0.12	0.12

CR, construct reliability; AVE, average variance extracted; ASV, average shared variance; MSV, mean shared variance.

The bi-factorial structure that we found could be the result of reverse coding (Woods, 2006). Even though the factor Difficulties in Empathizing revealed low construct reliability, we decided to keep the bi-factorial structure, since this is a pilot study of an adapted instrument to online contexts, which is quite different from the offline environment. Nonetheless, further studies are required to better assess the EQVC, and to better understand if the bi-factorial structure results from reverse coding, or from the characteristics of online contexts.

#### 4.2.2. Confirmatory factor analysis of the PMDCI

In order to confirm the initial structure suggested by the EFA of the scales from the PMDCI, various possible models were tested for the 5 scales (Supplementary Appendix Tables A.14–A.18). Hence, we attempted to test a model with all participants and no covariances (model 1), a model without outliers and no covariances (model 2) and a model without outliers and with covariances between the error terms (model 3).

Considering the *Locus Behavior* scale, the best model (model 3) presents several covariances between items (Supplementary Appendix Table A.14 and Supplementary Appendix Figure A.2). According to the literature (Jöreskog and Sörbom, 1984; Cole, 1987; Blunch, 2008), the factor model we opted for presented good reference values [χ^2^(25) = 9.638, χ^2^/*df* = 0.386, GFI = 0.991, AGFI =0.983, SRMR = 0.051, NFI = 0.975, PGFI =0.550, PNFI = 0.677].

As for the *Locus Agency* scale, model 3 which presents the covariances between two error terms of items (Supplementary Appendix Table A.15 and Supplementary Appendix Figure A.3) presented good reference values [χ^2^(8) = 1.233, χ^2^/*df* = 0.154, GFI = 0.997, AGFI =0.992, SRMR = 0.032, NFI = 0.987, PGFI =0.380, PNFI = 0.526], as according to the literature (Jöreskog and Sörbom, 1984; Cole, 1987; Blunch, 2008).

As for the *Locus Outcome* scale, we only assessed 2 models, since the Modification Indices did not indicate the need to covariate error terms of items (Supplementary Appendix Table A.16 and Supplementary Appendix Figure A.4), thus we only had model 1 with all participants, and model 2 without outliers. Model 2 presented good values [χ^2^(9) = 0.904, χ^2^/*df* = 0.100, GFI = 0.997, AGFI =0.993, SRMR = 0.028, NFI = 0.993, PGFI =0.427, PNFI = 0.596], as according to the literature (Jöreskog and Sörbom, 1984; Cole, 1987; Blunch, 2008). Nonetheless, Model 1 presented better validity in the population of study, since it has lower MECVI (Marôco, 2014).

Considering the *Locus Recipient* scale, model 3 presented the covariances between four error terms (Supplementary Appendix Table A.17 and Supplementary Appendix Figure A.5). According to the literature (Jöreskog and Sörbom, 1984; Cole, 1987; Blunch, 2008), the factor model we opted for presented good reference values [χ^2^(7) = 6.366, χ^2^/*df* = 0.909, GFI = 0.993, AGFI =0.979, SRMR = 0.042, NFI = 0.979, PGFI =0.331, PNFI = 0.457].

Finally, for the *Attribution scale*, model 3 presented the covariances between two error terms (Supplementary Appendix Table A.18 and Supplementary Appendix Figure A.6) revealed good reference values [χ^2^(26) = 1.198, χ^2^/*df* = 0.046, GFI = 0.992, AGFI =0.987, SRMR = 0.05, NFI =0.987, PGFI =0.573, PNFI = 0.713], according to the literature (Jöreskog and Sörbom, 1984; Cole, 1987; Blunch, 2008).

Despite the good fit of the selected models, PGFI did not present good values for all scales. It was below the cutoff of 0.6 (Blunch, 2008) in the Locus Agency, Outcome and Recipient and near the cutoff in the Locus Behavior and Attribution scale. Nonetheless, the other estimation procedures revealed good fit indices, supporting our model choice, as can be seen by comparing RMSEA and AIC. Also, all models chosen presented lower MECVI (Marôco, 2014), indicating better validity in the population of study, except for the Locus Outcome scale.

As can be seen in Supplementary Appendix Figures A.2–A.6, several relationships between each factor and corresponding items were lower than the cut-off value of 0.5 (Bandalos and Finney, 2010). Nevertheless, all unstandardized path coefficients were significant at *p* < 0.05. Moreover, the composite reliability scores ranged from 0.62 to 0.88, revealing medium to high construct reliability (Hair et al., 2014), as can be seen in Table 3. However, the AVE was low for Locus Behavior, Agency and Recipient and approximate of the 0.50 as indicated in the literature (Henseler et al., 2009) for Locus Outcome and Attributions. Thus, for the former scales, convergent validity was low, and for the later, convergent validity was almost adequate. Nonetheless, the Average Shared Variance (ASV) scores below the AVE scores (Hair et al., 2014) indicated good discriminant validity for all scales, except for Locus Outcome, of which the ASV could not be calculated, since this scale did not have correlation between error terms.

**Table 3:** Validity measures of Model 3 for all scales from the PMDCI.

Factors	Cronbach’s alpha	McDonald’s omega	CR	AVE	ASV	MSV
Locus behavior	0.76	0.76 [0.71, 0.79]	0.75	0.26	0.12	0.16
Locus agency	0.65	0.66 [0.57, 0.71]	0.62	0.24	0.07	0.07
Locus outcome	0.77	0.78 [0.72, 0.82]	0.80	0.42	N/A	0.00
Locus recipient	0.69	0.65 [0.54, 0.73]	0.68	0.30	0.18	0.22
Attributions	0.88	0.89 [0.80, 0.93]	0.88	0.45	0.10	0.10

CR, construct reliability; AVE, average variance extracted; ASV, average shared variance; MSV, mean shared variance. N/A, not available.

#### 4.2.3. Correlational study

In this investigation, we found that empathy in online contexts appeared to be divided in two factors (i.e., Difficulties in Empathizing and Self-efficacy regarding Empathy), and that Moral Disengagement with respect to cyberbullying situations was composed of 4 different *loci* (i.e., Behavior, Agency, Outcome and Recipient) and Attributions (for the definition of each scale/variable see Supplementary Appendix A.10). Thus, regarding the first research question, Difficulties in Empathizing was negatively and significantly correlated with Attributions (*r* = −0.135, *p* < 0.05) and 3 Locus of MD [Agent (*r* = −0.169, *p* < 0.01), Outcome (*r* = −0.218, *p* < 0.01), and Recipient (*r* = −0.142, *p* < 0.01)]. That is, the more difficulty participants had in empathizing, the less attributions and the three different Loci were used. However, with respect to self-efficacy in empathizing, it was not statistically significantly correlated with any variable. Considering the second research question, difficulties in empathizing was negatively and significantly correlated with gender (*r* = −0.114, *p* < 0.05), meaning that girls tended to have more difficulties in empathizing, and boys tended to have less. Additionally, gender was positively and significantly correlated with Attributions (*r* = 0.223, *p* < 0.01), Locus of Behavior (*r* = 0.174, *p* < 0.01), Locus of Agency (*r* = 0.226, *p* < 0.01), Locus of Outcome (*r* = 0.136, *p* < 0.05) and Locus of Recipient (*r* = 0.196, *p* < 0.01). This means that boys tended to use more attributions and MD Loci with regards to cyberbullying. Correlations can be found in Table 4.

**Table 4:** Correlations between EQVC and PMDCI.

Variable	Gender	Difficulties empathizing	Self-efficacy empathy	Attributions	Locus behavior	Locus agent	Locus outcome
Gender	**–**						
Difficulties empathizing	−0.114^*^	**–**					
Self-efficacy empathy	−0.041	−0.124^*^	–				
Attributions	0.223^**^	−0.135^*^	−0.072	–			
Locus behavior	0.174^**^	−0.072	0.000	0.410^**^	–		
Locus agent	0.226^**^	−0.169^**^	−0.075	0.234^**^	0.294^**^	–	
Locus outcome	0.136^*^	−0.218^**^	0.033	0.363^**^	0.580^**^	0.277^**^	–
Locus recipient	0.196^**^	−0.142^**^	0.026	0.404^**^	0.632^**^	0.393^**^	0.529^**^

*p < 0.05, **p < 0.01.

## 5. Discussion

Although investigating cyberbullying is crucial, it is difficult to assess adolescents’ view of this phenomenon since students tend to underrate their involvement (Francisco et al., 2015), which further demonstrates the importance of studying other related constructs, such as empathy and MD. That is, by understanding how these types of variables operate within the cyberbullying cycle, the more we are able to understand cyberbullying and its relationship with these variables. Thus, this investigation proposed a different perspective of these constructs, considering the specificities of the online world. Thus, we presented a preliminary study of two new instruments with respect to empathy and MD, considering that the characteristics of cyberspace can make right from wrong more difficult to distinguish (Marín-López et al., 2019), and have an impact on online interactions (Marín-López et al., 2020).

### 5.1. Empathy quotient in virtual contexts

Our proposed model of empathy in virtual contexts was highly distinct from the one initially proposed by Baron-Cohen and Wheelwright (2004) for face-to-face interactions. This was expected; since online contexts have some features that make feeling empathy difficult (Terry and Cain, 2016). Thus, instead of having three factors (i.e., cognitive empathy, emotional reactivity, and social skills) (Suler, 2004), EFA and CFA showed a bi-factorial structure. Therefore, the first factor refers to the difficulties in empathizing specifically in online contexts (by referring the term “difficulty” in most of the items) or not being able to understand something online. The second factor refers to self-efficacy beliefs regarding empathy, which according to Bandura (1997), refers to individuals’ beliefs regarding their capacity to control their own behavior and the environment that surrounds them, and specifically in this case, with respect to empathy.

This structure shares some similarities with the Portuguese short form of the EQ, since the factor Difficulties in Empathizing has the same 6 items as the Empathic Difficulties. Even though, two items had to be eliminated because of low discrimination, the fact that other study (Rodrigues et al., 2011) found a factor with the same structure gave us some support for our two-dimensional structure. Despite the bi-factorial structure of the EQVC, which could be a direct consequence of the reverse worded items, as well as careless respondents (Woods, 2006), if all the items of the first factor had already been aggregated together in other study (Rodrigues et al., 2011), we may suppose that they in fact, form a factor. Nonetheless, further investigation should be conducted, adding more (positively worded) items to this factor to reassess the bi-factorial structure and understand if it is specific to the online context.

As for the second factor, all items of Self-efficacy beliefs regarding Empathy refer to a capacity which is perceived by the participant (e.g., “I find it easy to put myself in someone else’s shoes online”). According to Bandura (2001, p.10) efficacy beliefs are the foundation of human agency, therefore the perceived self-efficacy to accomplish goals is more important than the actual capacity. These beliefs are the driving force to act, despite the difficulties that may arise in the course of action (Bandura, 2001). Thus, in the context of online empathy, it is of major importance that adolescents feel that they can deal with those situations, specifically considering online features that hamper empathy. Moreover, this structure informed us that in online contexts the different components of empathy (i.e., cognitive empathy), are not as relevant as the easiness/difficulty in feeling empathy, as well as the self-efficacy beliefs related to it.

Considering the results from this investigation, with respect to the factorial structure and reliability values, it seems important to continue this work of improving this instrument on empathy in virtual contexts, in order to understand whether the structure holds if more items are included, or if the instrument is analyzed with a different population, for example. Moreover, it would be interesting to test model invariance, in order to understand if the instrument behaves differently regarding boys and girls, separately. This would be important to test, since empathy is usually higher for girls (Jolliffe and Farrington, 2006). Moreover, it would also be interesting to evaluate the convergent validity, with other measures of MD in online interaction, as well as to assess discriminant validity with measures of empathy in virtual contexts.

### 5.2. Process moral disengagement in cyberbullying situations questionnaire

As for MD, instruments to address it related to cyberbullying situations have begun to appear (e.g., Bussey et al., 2015), but research on this topic remains a current concern (e.g., Paciello et al., 2020). For example, Bussey et al. (2015) addressed this issue in a general sense (i.e., “Cyberbullying annoying classmates is just teaching them a lesson”) or without specifying who the aggressor is (i.e., “If people give out their passwords to others, they deserve to be cyberbullied”). Items with this mixed approach made us question if the level of MD would be the same if participants put themselves in the place of aggressors or bystanders. Also, the qualitative research that led to the development of the instrument supported this idea, since adolescents did not use MD mechanisms only to legitimize cyberbullies’ actions, but also to approve cyber bystanders’ aggressive behavior (Francisco et al., 2022). Therefore, we decided to develop an instrument that could assess MD from the aggressors’ and bystanders’ perspectives. This distinctive feature allows us to understand the role of MD with respect to the aggressors’ and bystanders’ cyberbullying behavior, however, for the purpose of this study, only the bystander scale was analyzed.

With a different perspective, Marín-López et al. (2019) focused on Moral Justification, Diffusion of responsibility, Distortion of consequences and Attribution of blame. However, we wanted to capture the impact of MD mechanisms as a process. Thus, we chose to develop a measure that included all mechanisms, separated by locus, since the qualitative study showed that not all mechanisms have the same impact in explaining cyberbullying behavior (Francisco et al., 2022), and not all of them were mentioned (Figure 1). Moreover, for investigation purposes, some scales may prove to be more useful than others. Furthermore, we consider MD as a process; since this view provides a better understanding of how cyberbullying starts and how adolescents perpetuate this type of behavior, considering that some mechanisms may occur in specific timings of the cyberbullying cycle (Tillman et al., 2018).

Confirmatory Factor Analysis verified the unidimensionality of the five scales (i.e., 4 Locus and Attributions) of the Bystander perspective of the PMDCI. Future studies should evaluate the psychometric properties of the Aggressor’s perspective and compare it to the Bystander’s perspective. It would also be important to evaluate the convergent validity, with other measures of MD in online interaction, as well as to assess discriminant validity with measures of empathy in virtual contexts. Furthermore, it would also be very important, especially in terms of intervention, to understand if the role of the distinct loci differ according to different grade levels and participants’ age, because it is known that MD increases over the years in high school (Smith and Slonje, 2010) and severe cyberbullying incidents peak during middle adolescence (Festl et al., 2017).

### 5.3. Empathy online and moral disengagement in cyberbullying

With respect to the relationship between both constructs, we believe that when students felt more difficulties in empathizing, the need to resort to MD mechanisms to decrease moral self-sanctions lessened (Bandura, 2002). However, this does not mean that they would not get involved in cyberbullying situations. That is, if they did enter the cyberbullying cycle, since they had difficulties in empathizing, they would not use MD mechanisms, because they did not feel that the situation could transgress their moral standards. Considering gender issues, girls felt more difficulties in empathizing probably because they needed more social cues to do so (Suler, 2004; Runions and Bak, 2015). Even though they generally scored higher on empathy (Baron-Cohen and Wheelwright, 2004; Carrier et al., 2015), ICT may have brought them more challenges, especially considering that empathy can be effortful (Cameron et al., 2019), they may perceive more difficulties in empathizing. With respect to MD, we were expecting positive significant correlations regarding gender, since boys tended to express significantly higher levels of moral justification, euphemistic labeling, diffusion of responsibility, distortion of consequences and blaming the victim than girls (Thornberg and Jungert, 2014).

### 5.4. Limitations and future directions

This study has some limitations, among them the convenience sample (Marín-López et al., 2020), sample size (Gerdes et al., 2010), and age of participants (Barlett et al., 2016), therefore we cannot generalize findings. Additionally, self-report instruments can lead to false reporting and social desirability (Thornberg and Jungert, 2014), thus it would be interesting to compare adolescents’ results to peer reports (Garaigordobil, 2015). Also, procedures of data collection may not establish validity of the data (Gerdes et al., 2010), thus, comparison with objective data collected from ecologically valid contexts, would be important. Moreover, test–retest reliability would be important to better assess the instruments (Redondo and Herrero-Fernández, 2018).

### 5.5. Implications for practice

In terms of implications for practice, we believe the EQVC may provide some clues for intervention regarding the promotion of empathy in online contexts. Specifically, it can help identify which areas may be more prone to evoke some difficulties in feeling empathy when interacting virtually. Moreover, considering the importance of self-efficacy in goals and expectations (Bandura, 2001), it seems of extreme importance to stimulate and develop self-efficacy specific to online interactions, as well as to empower children and adolescents, so they can be able to persevere when deciding to act against cyberbullying events. Regarding MD, as Bandura et al. (1996) argued, the different mechanisms seem to differ in their contribution to detrimental conduct, hence the PMDCI allowed us to understand which MD mechanisms could interfere more with justifying cyberbullying behavior, and therefore, be an in-depth resource for interventions. That is, by providing information about the most common mechanisms used, this inventory can inform researchers and practitioners about what type of intervention can be developed within a specific population. Consequently, future interventions could be more accurate in terms of psychological needs, as well as more focused and shorter. These features may be important considering the difficulties that are often encountered with respect to the time available to work with children and adolescents beyond the school schedule. We believe that these versions of the EQVC and the PMDCI are promising instruments that can be further improved, and can also be used with other Portuguese-speakers (i.e., from Brazil and Angola, for example), however cultural differences may emerge. Moreover, we believe that these instruments can also be translated and adapted to other countries. Finally, the two instruments that resulted from this investigation can make an important contribution to understand the complex nature of cyberbullying to improve prosocial behavior online.

## Data availability statement

The datasets presented in this article are not readily available because the Portuguese National Commission of Data Protection and the Deontology Committee of the researchers’ institution do not allow the availability of the datasets. The data that supports the findings of this study are available in the Supplementary material of this article. Requests to access the datasets should be directed to sofifrancisco@gmail.com.

## Ethics statement

The studies involving human participants were reviewed and approved by Deontology Committee of the Faculty of Psychology University of Lisbon. Written informed consent to participate in this study was provided by the participants’ legal guardian/next of kin.

## Author contributions

SF designed and executed the study, analyzed the data, and wrote the manuscript. PC assisted with the design, collaborated with the data analyses, and the writing of the study. AV assisted with the design, execution and writing of the study, collaborated with the editing of the final manuscript. NP assisted with writing and the editing of the final manuscript. All authors approved the final version of the manuscript for submission.

## Funding

This work was supported by the Foundation for Science and Technology of the Science and Education Ministry of Portugal through a PhD grant (SFRH/BD/130982/2017), a Project grant (PTDC/PSI-GER/1918/2020) and through the Research Center for Psychological Science of the Faculty of Psychology, University of Lisbon (CICPSI; UIDB/04527/2020 and UIDP/04527/2020).

## Conflict of interest

The authors declare that the research was conducted in the absence of any commercial or financial relationships that could be construed as a potential conflict of interest.

## Publisher’s note

All claims expressed in this article are solely those of the authors and do not necessarily represent those of their affiliated organizations, or those of the publisher, the editors and the reviewers. Any product that may be evaluated in this article, or claim that may be made by its manufacturer, is not guaranteed or endorsed by the publisher.

## Footnotes

^1^Unstandardized path coefficients and corresponding significant statistics were not available for ULS, thus, we present values from the ML with Bollen-Stine Bootstrap.
