# Supplementary material for: Measuring empathy online and moral disengagement in cyberbullying
Source: Front Psychol. 2023 Apr 27;14:1061482. doi: 10.3389/fpsyg.2023.1061482 (PMC10172580; doi:10.3389/fpsyg.2023.1061482)
Supplement: Supplementary file 1 [file Data_Sheet_1.pdf]

Supplementary Material

Appendix A.1

Initial adaptation of the EQVC

We adapted the Portuguese version of the EQ-short form (Rodrigues et al., 2011) to online contexts, which includes 22 items on a 4-point scale, scored from 0 to 2: 0 for *strongly disagree* and *slightly disagree* (i.e., non-empathic response), 1 for *slightly agree* and 2 for *strongly agree* (i.e., empathic response) (Baron-Cohen et al., 2003; Baron-Cohen & Wheelwright, 2004).Six items were reverse coded (3, 4, 5, 7, 11 and 17): "*strongly disagree*" responses were scored 2 points and "*slightly disagree*" responses are scored 1 point. Higher scores reflect students that reported a higher level of empathy. The response scale was modified, from 1 to 4 points (*totally disagree* to *totally agree*), because the questionnaire was embedded in a larger inventory with this type of response, and we wanted to reflect homogeneity in the Likert scales used, to decrease confusion in the type of responses.

The final stage of the instrument adaptation was its facial validity, with a 9th grade class. All 22 items were presented to the class, as well as a few questions regarding the vocabulary, their understanding of the items and suggestions students wish to make. Very small changes were made to the items.

Table A.1

Original and translated items from the EQ-Short Form and items adapted to the virtual context (EQVC)

Original Item	Portuguese Version Items (EQ-Short form)	EQVC Items
1. I can easily tell if someone else wants to enter a conversation.	Eu consigo, facilmente, dizer se alguém quer entrar numa conversa.	1. Consigo perceber facilmente se alguém quer entrar numa conversa online.
6.I really enjoy caring for other people	Eu gosto realmente de me preocupar com as outras pessoas.	2. Gosto mesmo de me preocupar com as outras pessoas online.
8. I find it hard to know what to do in a social situation	Eu considero difícil saber o que fazer numa situação social.	3. Tenho dificuldade em saber o que fazer numa rede social.
14. I often find it difficult to judge if something is rude or polite.	Frequentemente tenho dificuldades em julgar se algo é ofensivo ou simpático.	4. Muitas vezes tenho dificuldade em perceber se algo online é ofensivo ou simpático.
15. In a conversation, I tend to focus on my own thoughts rather than on what my listener might be thinking.	Numa conversa, eu tendo a focar-me nos meus pensamentos em vez de me focar no que o meu ouvinte possa estar a pensar.	5. Numa conversa online, eu concentro-me mais nos meus pensamentos do que naquilo em que o outro possa estar a pensar.
19. I can pick up quickly if someone says one thing but means another.	Eu consigo perceber rapidamente quando alguém diz uma coisa mas quer dizer outra.	6. Consigo perceber depressa se alguém quer dizer uma coisa online, mas diz outra.
21. It is hard for me to see why some things upset people so much.	Para mim, é complicado ver porque algumas coisas chateiam tanto as pessoas.	7. Tenho dificuldade em perceber porque é que algumas coisas chateiam tanto as pessoas online.
22. I find it easy to put myself in somebody else's shoes.	É fácil, para mim, colocar-me no lugar de outra pessoa.	8. É fácil colocar-me no lugar de outra pessoa online.
25. I am good at predicting how someone will feel	Eu sou bom a predizer como alguém se irá sentir.	9. Consigo prever como alguém se irá sentir online.
26. I am quick to spot when someone in a group is feeling awkward or uncomfortable.	Eu vejo com facilidade quando alguém, num grupo, se está a sentir embaraçado ou desconfortável.	10. Percebo depressa quando alguém, num grupo online, se sente esquisito ou desconfortável.
29. I can't always see why someone should have felt offended by a remark.	Nem sempre consigo perceber porque alguém se terá sentido ofendido por um reparo.	11. Nem sempre consigo perceber porque alguém terá ficado magoado com uma crítica online.
35. I don't tend to find social situations confusing	Não tendo a achar as situações sociais confusas.	12. Normalmente não acho as redes sociais confusas.
36. Other people tell me I am good at understanding how they are feeling and what they are thinking.	As outras pessoas dizem-me que sou bom a perceber como elas se sentem ou o que estão a pensar.	13. As outras pessoas que estão online dizem-me que consigo compreender como elas se sentem e o que estão a pensar.

Table A.1 (continuation)

Original and translated items from the EQ-Short Form and items adapted to the virtual context (EQVC)

Original Item	Portuguese Version Items (EQ-Short form)	EQVC Items
41. I can easily tell if someone else is interested or bored with what I am saying.	Eu consigo perceber com facilidade quando alguém está interessado ou aborrecido com o que estou a dizer.	14. Consigo perceber facilmente se alguém está interessado ou aborrecido com o que estou a dizer online.
43. Friends usually talk to me about their problems as they say that I am very understanding.	Normalmente os meus amigos falam-me dos seus problemas e dizem que sou muito compreensivo.	15. Os meus amigos online contam-me os seus problemas frequentemente porque dizem que sou muito compreensivo(a).
44. I can sense if I am intruding, even if the other person doesn't tell me.	Eu percebo quando estou a ser intrometido mesmo que a outra pessoa não mo diga.	16. Percebo quando estou a ser intrometido(a) online mesmo se a outra pessoa não mo diz.
48. Other people often say that I am insensitive, though I don't always see why.	Frequentemente as outras pessoas dizem que sou insensível, se bem que nem sempre percebo porquê.	17. As outras pessoas online dizem que sou insensível frequentemente, mas nem sempre percebo porquê.
52. I can tune into how someone else feels rapidly and intuitively.	Eu consigo sintonizar-me com o que os outros sentem, rapidamente e intuitivamente.	18. Consigo aperceber-me de como alguém se sente, rápida e intuitivamente, quando estou online.
54. I can easily work out what another person might want to talk about.	Eu consigo descobrir rapidamente o assunto sobre o que outra pessoa quer falar.	19. Consigo perceber facilmente sobre o que outra pessoa quer falar online.
55. I can tell if someone is masking their true emotion	Eu consigo perceber quando outra pessoa está a disfarçar os seus verdadeiros sentimentos.	20. Consigo perceber quando outra pessoa está a disfarçar os seus verdadeiros sentimentos online.
58. I am good at predicting what someone will do	Eu sou bom a prever o que outra pessoa irá fazer.	21. Consigo prever o que outra pessoa irá fazer online.
59. I tend to get emotionally involved with a friend's problems.	Eu tendo a envolver-me emocionalmente com os problemas dos meus amigos.	22. Envolvo-me facilmente de forma emocional nos problemas dos meus amigos online.

Appendix A.2

Initial construction of the PMDCI

Through the in-depth semi structure interviews with scenarios, we intended to understand what motivates the aggressor to engage in cyberbullying behavior ("What do you think led the author to publish this post?"), what motivates and influences bystanders' behavior ("What do you think about these comments?), and finally, we intended to place the participant in the perspective of the bystander in different scenarios ("Would you react to these comments? How?), to comprehend his/her point of view regarding the cyberbullying scenarios.

The following mixed content analysis allowed us to better understand how adolescents perceive cyberbullying episodes, including both aggressor's and bystander's behavior. The coding units for this analysis were adolescents' written propositions with meaning (total of 396 propositions were analyzed). When categorizing the propositions, we were deductively guided by the SCT of Moral Agency (Bandura, 2002). Thus, deductive content analysis was based on the following mechanisms, which are organized in four loci. Firstly, locus of behavior, where immoral conduct is transformed into fair or justifiable conduct through *Moral Justification,* which allows harmful conductto be reconstructed as serving social or moral intentions; *Euphemistic Labeling,* which decreases the severity of a behavior by naming it as less severe behavior, and through *Advantageous Comparison,* by contrasting with other behavior, and therefore, harmful acts are considered morally correct. Secondly, there is locus of agency, where the perpetrator's agentic role is minimized in detrimental conduct through the *Displacement of Responsibility,* in which individuals view their actions as resulting from others' orders, and the *Diffusion of Responsibility,* which enables individuals to divide the responsibility for an action among a group. The third locus is based on the

outcome of the behavior, where moral control is faded, depending on the outcome of one's actions, through the *Distortion of consequences*, which inhibits the activation of self-censure, because one's' conduct is overlooked, minimized, distorted, or disregarded. Lastly, the locus of the recipient, which implies self-exonerating one's own actions by *Dehumanizing* the victims, that is, depriving them of their human qualities and by *Attributing* them the *Blame* and allowing the aggressor to justify their actions, and considering the victim responsible for his/her suffering (Bandura, et al., 1996; Bandura, 2002).

However, other categories emerged through inductive reasoning. Resulting from this analysis, several MD mechanisms as well as other attributions for the cyberbullying behaviors have emerged, as can be seen in Figure 1 in the main text.

After a thorough study of all categories and corresponding propositions, we started developing the questionnaire itself, following the steps mentioned below:

Step 1: Generation of item pool

The first step was to create a pool of item depicting the categories that emerged from the content analysis, both regarding cyberbullying behavior and motives for not intervening in these situations, mainly because interviewed students used MD mechanisms to justify this type of inaction (total of 67 items). For conducting this task, several brainstorming meetings took place, to convert MD mechanisms into cyberbullying situations. The items referring to the MD mechanisms that emerged from the content analysis were based on Moral Disengagement Scale (Bandura et al., 1996). The items regarding other attributions for cyberbullying behavior, as well as the non-intervention items were based on the verbalizations from the interviewed participants. This first pool of items referred to cyberbullying behavior in general, not focusing on the aggressors' nor bystanders' behavior. However, we concluded that the items need to

be more homogeneous, and we decided to develop another pool of items (120 items) including items with three different perspectives: aggressors' and bystanders' cyberbullying behavior (e.g. "If I make fun of someone else, it is not as bad as hitting him/her", "If I see someone hitting another person, I think it is worse than making fun of her/him online", respectively) and cyberbullying acts in general, without specifying who is the aggressor (e.g. "Making fun of someone online is not as bad as hitting him/her"). Most of the items from the second pool were improved items from the previous pool. Despite the quality of the items generated, in order to include all MD and cyberbullying behavior, the team decided to eliminate all items that referred to the cyberbullying acts in general, with the main objective of not burden the participants. Moreover, since it would be impossible to comprise all cyberbullying behavior in one instrument and we wanted the instrument to be the more accurate as possible, we based our items in three types of behavior: insults, making fun and sexual harassment since the insults is the more frequent (Francisco et al., 2015), making fun is considered the less harmful and sexual harassment was considered the most harmful. Thus, this pool was composed by 78 items, with 3 items for each behavior and each perspective, for both MD mechanisms and attributions, and further 12 items for non-intervention motives.

Step 2: Experts' Evaluation

The item pool was evaluated by an expert panel familiar to the cyberbullying phenomenon as well as the Bandura's MD mechanisms. Items were evaluated according to 4 categories: sufficiency, clarity, coherence, and relevance (Escobar-Pérez & Cuervo-Martínez, 2008). After discussion and validation by experts of all the items, we decided to reduce this pool of items to the final version, including 84 items.

Step 3: Face validity by students

The final step in developing the PMDCI was its face validity by students. We gathered a 9th-grade class and presented them all items of the inventory along with a few questions regarding the adequacy of the vocabulary if they understood the items and possible suggestions. After analysis of students' feedback, a few changes were made in some items, specifically in terms of vocabulary.

Appendix A.3

The PMDCI structure

The inventory includes 3 demographic questions (age, sex, and school year) and 84 Likert Scale. It comprises 2 questionnaires, considering the aggressor's perspective (36 items) and the bystander's perspective (36 items), both about possible cyberbullying situations. Twenty-four items of both questionnaires were developed considering the eight MD mechanisms from Bandura's theory (i.e., 3 items for each mechanism: Moral justification, Advantage comparison, Euphemistic Labeling, Displacement of responsibility, Diffusion of responsibility, Distortion of consequences, Blaming the victim, and Dehumanization). Additionally, 3 items for Devaluation of behavioral intention, and 9 items for the attribution category (3 items for each subcategory: Absence of affective bonding, interpersonal relationships, and personal factors). Finally, it also presents a Non-Intervention scale, comprising 12 items that intend to assess the motives that lead to this passive response, in the perspective of bystanders. Specifically, the scale comprehends: No need for intervention, Absence of relationship with those involved in the situation, Self-protection in the face of the situation, Low self-efficacy beliefs, Diffusion and Displacement of responsibility. The last two were MD mechanisms that adolescents frequently used to discard their place in intervention.

Table A.2

Items from the Locus Behavior Scale from the PMDCI – Portuguese version

Moral Disengagement mechanism	Items from the aggressors' perspective	Items from the bystanders' perspective
Moral Justification	1.Não faz mal se eu assediar sexualmente alguém online, se essa pessoa me rejeitou.<br>2.Não há problema em gozar com alguém online que maltratou os meus amigos.<br>3.Não acho mal se eu insultar uma pessoa online que maltratou a minha família.	1.Não há problema se eu vir uma pessoa assediar sexualmente online outra que a rejeitou.<br>2.Não faz mal se eu vir uma pessoa gozar com outra pessoa online que maltratou os(as) seus(suas) amigos(as).<br>3.Não há problema se eu vir alguém a insultar outra pessoa online que maltratou a sua família.
Euphemistic labelling	1.Se eu assediar sexualmente alguém online é só uma brincadeira<br>2.Se eu gozar com outra pessoa online, estou só a brincar.<br>3.Se eu insultar outra pessoa online para mim é normal.	1.Se eu vir alguém a assediar sexualmente outra pessoa online é só uma brincadeira.<br>2.Se vir alguém a gozar com outra pessoa online, acho que ele/a está só a brincar.<br>3.Se eu vir alguém a insultar outra pessoa online, acho que isso é normal.
Advantage Comparison	1.Se eu assediar sexualmente alguém online não é tão mau como persegui-la pessoalmente.<br>2.Se eu gozar com outra pessoa online não é tão mau como bater-lhe.<br>3.Se eu insultar alguém online não é tão sério como bater-lhe.	1.Se eu vir alguém a assediar sexualmente outra pessoa online, acho que não é tão mau como se a perseguisse pessoalmente.<br>2.Se eu vir alguém a gozar com outra pessoa online, acho que não é tão mau como bater-lhe.<br>3.Se eu vir alguém a insultar outra pessoa online, acho que não é tão sério como bater-lhe.

Table A.3

Items from the Locus Agency Scale from the PMDCI– Portuguese version

Moral Disengagement mechanism	Items from the aggressors' perspective	Items from the bystanders' perspective
Displacement of responsibility	1.Se eu assediar sexualmente alguém online, a responsabilidade é dos meus(minhas) amigos(as) porque me influenciaram.<br>2.Se eu gozar com outras pessoas online, a responsabilidade é dos meus(minhas) amigos(as) porque me influenciaram.<br>3.Se eu insultar alguém online, a responsabilidade é dos meus(minhas) amigos(as) porque me influenciaram.	1.Se eu vir alguém a assediar sexualmente outra pessoa online, a responsabilidade é dos seus(suas) amigos(as) porque o(a) influenciaram.<br>2.Se eu vir alguém a gozar com outra pessoa online, a responsabilidade é dos seus(suas) amigos(as) porque o(a) influenciaram.<br>3.Se eu vir alguém a insultar outra pessoa online, a responsabilidade é dos seus(suas) amigos(as) porque o(a) influenciaram.
Diffusion of responsibility	1.Se eu assediar sexualmente alguém online, não devo ser responsabilizado porque todos no grupo fazem isso.<br>2.Se eu gozar com alguém online, não devo ser responsabilizado porque todos os jovens fazem isso.<br>3.Se eu insultar alguém online, não devo ser responsabilizado porque não sou o único(a).	1.Se eu vir alguém a assediar sexualmente outra pessoa online,acho que essa pessoa não deve ser responsabilizada porque todos no grupo fazem isso.<br>2.Se eu vir alguém a gozar com outra pessoa online, acho que essa pessoa não deve ser responsabilizada porque todos os jovens fazem isso.<br>3.Se eu vir alguém a insultar outra pessoa online, acho que essa pessoa não deve ser responsabilizada porque não é a única.

Table A.4

Items from the Locus Outcome Scale from the PMDCI– Portuguese version

Moral Disengagement mechanism	Items from the aggressors' perspective	Items from the bystanders' perspective
Devaluation of behavioral intentions	1.Se eu assediar sexualmente alguém na internet, não é com intenção de fazer nada.<br>2.Se eu gozar com alguém online, não é para magoar.<br>3.Se eu insultar alguém online, não é para magoar.	1.Se eu vir alguém a assediar sexualmente outra pessoa online, acho que não é com intenção de fazer nada.<br>2.Se eu vir alguém a gozar com outra pessoa online, acho que não é para magoar.<br>3.Se eu vir alguém a insultar outra pessoa online, acho que não é para magoar.
Distortion of consequences	1.Se eu assediar sexualmente alguém online, ele/ela vai achar que é porque estou interessado(a) nele(a).<br>2.Se eu gozar com alguém online, eu acho que ele/ela não vai levar a mal.<br>3.Se eu insultar alguém online, eu acho que não a vou magoar.	1.Se eu vir alguém a assediar sexualmente outra pessoa online, essa pessoa vai achar que é porque está interessado(a) nela(e).<br>2.Se eu vir alguém a gozar com outra pessoa online, acho que ele/ela não vai levar a mal.<br>3.Se eu vir alguém a insultar outra pessoa online, eu acho que isso não a vai magoar.

Table A.5

Items from the Locus Recipient Scale from the PMDCI – Portuguese version

Moral Disengagement mechanism	Items from the aggressors' perspective	Items from the bystanders' perspective
Attribution of blame	1.Se eu assediar sexualmente alguém online, é porque essa pessoa mostrou demais nas fotos que postou.<br>2.Se eu gozar com alguém online, é porque essa pessoa se expôs demais.<br>3.Se eu insultar alguém online, é porque essa pessoa mereceu.	1.Se eu vir alguém a assediar sexualmente outra pessoa online, é porque essa pessoa mostrou demais nas fotos que postou.<br>2.Se eu vir alguém a gozar com outra pessoa online, é porque essa pessoa se expôs demais.<br>3.Se eu vir alguém a insultar outra pessoa online, é porque essa pessoa mereceu.
Dehumanization	1.Não há problema se eu assediar sexualmente alguém online, se essa pessoa se portou como um/a porco/a.<br>2.Não faz mal se eu gozar com outra pessoa online, se essa pessoa é uma besta.<br>3.Não há problema se eu insultar outra pessoa online, se essa pessoa é um mostro.	1.Não há problema se eu vir alguém a assediar sexualmente alguém online, se essa pessoa se portou como um/a porco/a.<br>2.Não faz mal se eu vir alguém a gozar com outra pessoa online, se essa pessoa é uma besta.<br>3.Não há problema se eu vir alguém a insultar outra pessoa online, se essa pessoa é um mostro.

Table A.6

Items from the Attributions Scale from the PMDCI– Portuguese version

Moral Disengagement mechanism	Items from the aggressors' perspective	Items from the bystanders' perspective
Absence of affective bonding	1.Não há problema se eu assediar sexualmente alguém online porque não gosto dessa pessoa.<br>2.Não faz mal se eu gozar com alguém online porque não conheço essa pessoa.<br>3.Não há problema se eu insultar alguém online porque não gosto dessa pessoa.	1.Não faz mal se eu vir alguém a assediar sexualmente outra pessoa online porque não gosta dela.<br>2.Não faz mal se eu vir alguém a gozar com outra pessoa online porque não se conhecem.<br>3.Não há problema se eu vir alguém a insultar outra pessoa online porque não gosta dela.
Aggressors' factors	1.Não faz mal se eu assediar sexualmente alguém online porque sinto raiva dessa pessoa.<br>2.Não faz mal se eu gozar com alguém online se faço isso para me sentir melhor comigo próprio.<br>3.Não há problema se eu insultar alguém online para me sentir superior a essa pessoa.	1.Não há problema se eu vir alguém a assediar sexualmente outra pessoa online porque sente raiva dela.<br>2.Não faz mal se eu vir alguém a gozar com outra pessoa online para se sentir melhor consigo próprio.<br>3.Não faz mal se eu vir alguém a insultar outra pessoa online para se sentir superior a essa pessoa.
Interpersonal relationships	1.Não há problema se eu assediar sexualmente alguém se me quero integrar num grupo online onde isso é normal.<br>2.Não faz mal se eu gozar com alguém online se faço isso para não ser eu o próximo alvo.<br>3.Não há problema se eu insultar alguém online para me afastar dessa pessoa.	1.Não faz mal se eu vir alguém a assediar sexualmente outra pessoa porque se quer integrar num grupo online e isso é normal nesse grupo.<br>2.Não faz mal se eu vir alguém a gozar com outra pessoa online para não ser ela o próximo alvo.<br>3.Não há problema se eu vir alguém a insultar outra pessoa online para se afastar dela.

Appendix A.4

Participants

Most students were Portuguese (89.3%) and other students were mainly from other European countries (e.g., Romania, Italy, Switzerland) and from African countries (e.g., São Tomé and Príncipe, Angola, Cape Verde), and other nationalities were from South America (e.g., Colombia and Venezuela) and from Asia (e.g., Japanese, Chinese).

Appendix A.5

Exploratory evidence of the EQVC

Before proceeding to the EFA several procedures needed to be implemented. Specifically, for the correlation matrix, we used polychoric correlations which are recommended for polytomous items with asymmetric univariate distribution of ordinal items, according to the literature (Brown, 2006; Muthén & Kaplan, 1985).

In order to retain the appropriate number of factors and considering that the Cattel Scree test is subjective and an unreliable procedure (Zwick & Velicer, 1986), especially in exploratory studies, where the number of factors is not known (Kanyongo, 2005), we used Horn Parallel analyses (O’Connor, 2000), since it is one of the more precise methods for factor estimation (Hayton et al., 2004). The Optimal Implementation of Parallel Analysis (Timmerman & Lorenzo-Seva, 2011) suggested that two factors should be extracted. And, based on the excess kurtosis, we used the Unweighted Least Squares (ULS) method for factor extraction, since this method does not rely on distributional assumptions (Jöreskog & Sörbom, 1982).

Appendix A.6

Comparison between bifactorial and unifactorial models

Considering that the first factor included all items that were reverse worded, we attempted to test a unifactorial model to see if the model fit of this model was better than the bifactorial model with the separate “method factor” (Woods, 2006). Results

showed that the bifactorial model had better fit than the model with all items in a single factor (Table A.9). Moreover, the decision for considering both factors (Difficulties in empathizing and Self-efficacy beliefs regarding empathy) was not just depending on the fit of the models but also on literature, that considers that empathy can be difficult by the specificities of the online context (Pfetsch, 2017; Suler, 2004). Furthermore, we decided to keep the bifactorial model since the percentage of explained variance was higher.

Appendix A.7

Reliability

Table A.3 presents Cronbach's alpha since it is one of the most used reliability measures, however it can be inaccurate when used for ordinal scales. Thus, in the text we only present McDonald's Omega since it has less risk of overestimation or underestimation of reliability(Dunn et al., 2013). Nonetheless, the results are very similar between methods.

Table A.7

Descriptive Statistics and Polychoric Correlations of the EQVC

Variable	*Mean (SD)*	Skewness	Kurtosis	Polychoric correlations																				
				1	2	3	4	5	6	7	8	9	10	11	12	13	14	15	16	17	18	19	20	21
1	2.88(1.02)	-0.563	-0.786																					
2	2.55(1.05)	-0.114	-1.164	0.40																				
3	3.21(1.00)	-0.956	-0.370	0.05	-0.08																			
4	2.80(1.09)	-0.380	-1.158	-0.08	-0.20	0.27																		
5	2.74(1.08)	-0.333	-1.161	-0.13	-0.11	0.19	0.18																	
6	2.63(1.07)	-0.146	-1.207	0.41	0.37	-0.10	-0.17	-0.23																
7	2.62(1.05)	-0.083	-1.183	-0.16	-0.29	0.24	0.31	0.32	-0.19															
8	2.44(1.08)	0.052	-1.257	0.18	0.48	-0.11	-0.21	-0.12	0.35	-0.20														
9	2.35(1.06)	0.123	-1.201	0.28	0.41	-0.13	-0.18	-0.26	0.49	-0.30	0.60													
10	2.69(1.04)	-0.224	-1.128	0.37	0.47	-0.18	-0.23	-0.17	0.49	-0.28	0.38	0.52												
11	2.80(.95)	-0.248	-0.934	-0.09	-0.21	0.33	0.34	0.36	-0.14	0.47	-0.16	-0.25	-0.19											
12	2.65(1.08)	-0.246	-1.194	0.29	0.28	0.00	-0.08	-0.25	0.36	-0.32	0.15	0.40	0.40	-0.25										
13	2.34(1.08)	0.155	-1.244	0.35	0.39	-0.19	-0.19	-0.15	0.47	-0.36	0.44	0.50	0.56	-0.31	0.42									
14	2.66(1.08)	-0.197	-1.220	0.23	0.48	-0.22	-0.29	-0.21	0.47	-0.34	0.42	0.47	0.65	-0.25	0.46	0.57								
15	2.60(1.10)	-0.136	-1.299	0.29	0.34	-0.13	-0.01	-0.13	0.34	-0.31	0.34	0.45	0.61	-0.24	0.48	0.61	0.54							
16	2.74(1.11)	-0.361	-1.205	0.26	0.32	-0.16	-0.23	-0.25	0.40	-0.29	0.33	0.37	0.53	-0.32	0.46	0.52	0.58	0.60						
17	3.07(1.01)	-0.681	-0.767	-0.02	-0.13	0.43	0.33	0.26	-0.17	0.38	-0.10	-0.17	-0.15	0.44	-0.17	-0.15	-0.22	-0.21	-0.27					
18	2.44(1.04)	0.045	-1.156	0.29	0.37	-0.21	-0.20	-0.21	0.53	-0.35	0.40	0.59	0.56	-0.24	0.53	0.61	0.62	0.54	0.55	-0.27				
19	2.55(1.07)	-0.166	-1.207	0.38	0.40	-0.25	-0.11	0.20	0.50	-0.24	0.39	0.49	0.56	-0.24	0.43	0.51	0.62	0.57	0.54	-0.36	0.64			
20	2.59(1.07)	-0.086	-1.220	0.22	0.51	-0.25	-0.19	-0.16	0.50	-0.32	0.39	0.42	0.49	-0.18	0.42	0.53	0.66	0.51	0.52	-0.16	0.58	0.63		
21	2.06(1.02)	0.491	-0.942	0.23	0.34	-0.21	-0.24	-0.27	0.49	-0.25	0.43	0.55	0.42	-0.29	0.26	0.49	0.45	0.32	0.43	-0.37	0.55	0.56	0.56	
22	2.32(1.05)	0.189	-1.152	0.13	0.43	-0.24	-0.21	-0.11	0.33	-0.33	0.37	0.47	0.47	-0.35	0.29	0.50	0.51	0.57	0.38	-0.31	0.48	0.44	0.46	0.49

Table A.8

Exploratory Factor Analysis Parameters and Multidimensional Normal-ogive Graded Response Model parameters of the EQVC

Variable	Structure Coefficients		Slope and threshold parameters					
	Difficulty	Self-efficacy	a_1	a_2	*MDISC*	d_1	d_2	d_3
3. I find it hard to know what to do on a social network.	**0.555**	0.061	0.652	0.071	0.656	-1.609	-0.817	-0.126
4. I often find it hard to know if something is offensive or polite online.	**0.480**	-0.010	0.548	-0.012	0.548	-1.106	-0.361	0.466
5. In an online conversation, I tend to focus more on my own thoughts rather than on what the other person might be thinking.	**0.383**	-0.076	0.424	-0.084	0.432	-0.997	-0.325	0.569
7. I find it hard to see why some things bother people so much online.	**0.502**	-0.162	0.630	-0.204	0.662	-1.193	-0.094	0.821
11. I can't always see why someone could have felt hurt by a remark online.	**0.708**	0.025	0.986	0.035	0.986	-1.832	-0.408	0.820
17. Other people say I'm often insensitive online, but I don't always understand why.	**0.738**	0.087	1.027	0.121	1.034	-1.869	-0.768	0.164
1. I can easily tell if someone wants to enter a conversation online.	0.191	**0.523**	0.214	0.586	0.624	-1.206	-0.577	0.509
2. I really enjoy caring for other people online.	0.051	**0.612**	0.063	0.755	0.758	-0.998	-0.132	0.980
6. I can quickly tell if someone wants to say one thing online but says something else.	0.100	**0.695**	0.131	0.912	0.922	-1.183	-0.169	0.825
8. I find it easy to put myself in someone else's shoes online.	0.043	**0.578**	0.052	0.696	0.698	-0.820	0.052	0.973
9. I can predict how someone will feel online.	0.011	**0.699**	0.015	0.971	0.971	-0.836	0.149	1.343
10. I notice quickly when someone feels awkward or uncomfortable in an online group.	0.096	**0.799**	0.145	1.215	1.223	-1.497	-0.312	0.915
12. I don't usually find social networks confusing.	0.018	**0.575**	0.022	0.697	0.697	-0.998	-0.275	0.777
13. Other people tell me that I can understand what they feel and what they are thinking online.	0.016	**0.758**	0.025	1.145	1.145	-0.852	0.195	1.386
14. I can easily tell if someone is interested or annoyed with what I'm saying online.	-0.033	**0.763**	-0.052	1.222	1.223	-1.417	-0.276	0.944
15. My online friends often tell me their problems because they say I'm very understanding.	0.077	**0.750**	0.110	1.069	1.075	-1.110	-0.168	0.877
16. I notice when I'm being nosy online even if the other person doesn't tell me.	-0.087	**0.637**	-0.120	0.878	0.886	-1.155	-0.435	0.658
18. I can tell how someone is feeling, quickly and intuitively, when I'm online.	-0.015	**0.781**	-0.024	1.271	1.272	-1.198	0.070	1.467
19. I can easily tell what someone wants to talk about online.	-0.013	**0.756**	-0.021	1.171	1.171	-1.140	-0.250	1.229
20. I can tell when someone else is disguising their true feelings online.	0.022	**0.754**	0.033	1.126	1.126	-1.298	-0.112	0.997
21. I can predict what someone else will do online.	-0.188	**0.554**	-0.254	0.748	0.789	-0.426	0.582	1.679
22. I easily get emotionally involved in my online friends' problems.	-0.189	**0.531**	-0.249	0.699	0.742	-0.793	0.227	1.297
Eigenvalues	1.42	8.04						
Cronbach's Alpha α	.68	.91						

Table A.9

Unifactorial and bifactorial model parameters of the Exploratory Factor Analysis of EQVC

	Mardia's Coefficient		Kaiser-Meyer-Olkin	Bartlett Sphericity	% Explained Variance	GFI	CFI	RMSR	RMSEA	α	ω	
	S	*K*										
Unifactorial Model	78.41 < 22(22 + 2) = 528	605.06> 22(22+2) = 528	.89	χ^2_{231} = 2543.4 (*p* < .001)	39%	.96	.98	.083	0.028	.79	.82	
Bifactorial Model	78.41 < 22(22 + 2) = 528	605.06> 22(22+2) = 528	.89	χ^2_{231} = 2543.4 (*p* < .001)	48%	.98	1.00	.057	0.028	.68 .91	.68 [.58, .74]	.91 [.88, .93]

Table A.10

Descriptive Statistics and Polychoric Correlations of the Five Scales of the PMDCI

Variables	Mean(*SD*)	Skewness	Kurtosis	Polychoric correlations							
				1	2	3	4	5	6	7	8
Locus Behavior											
Item 1	1.454(.82)	1.782	2.173								
Item 2	1.467(.81)	1.661	1.780	0.67							
Item 3	1.742(.96)	1.046	-0.074	0.54	0.67						
Item 4	1.389(.76)	1.975	3.069	0.79	0.83	0.63					
Item 5	1.655(.88)	1.195	0.465	0.59	0.66	0.60	0.70				
Item 6	1.607(.94)	1.459	0.966	0.49	0.60	0.55	0.55	0.54			
Item 7	1.541(.85)	1.457	1.075	0.62	0.74	0.52	0.70	0.59	0.64		
Item 8	1.629(.90)	1.242	0.447	0.51	0.58	0.54	0.60	0.53	0.59	0.77	
Item 9	1.607(.95)	1.356	0.556	0.58	0.63	0.43	0.63	0.54	0.70	0.59	0.64
Locus Agency											
Item 1	1.712(1.01)	1.184	0.061								
Item 2	1.467(.82)	1.783	2.284	0.65							
Item 3	1.507(.84)	1.538	1.289	0.54	0.74						
Item 4	1.620(.89)	1.238	0.432	0.58	0.60	0.71					
Item 5	1.546(.87)	1.465	1.053	0.53	0.55	0.69	0.66				
Item 6	1.507(.83)	1.505	1.197	0.57	0.66	0.74	0.64	0.86			
Locus Outcome											
Item 1	1.376(.77)	2.182	3.990								
Item 2	1.485(.87)	1.808	2.219	0.77							
Item 3	1.489(.79)	1.558	1.610	0.74	0.75						
Item 4	1.550(.86)	1.501	1.268	0.61	0.55	0.78					
Item 5	1.480(.81)	1.609	1.594	0.70	0.69	0.86	0.79				
Item 6	1.472(.81)	1.746	2.251	0.71	0.73	0.88	0.74	0.85			
Locus Recipient											
Item 1	1.777(.99)	0.916	-0.492								
Item 2	1.725(.95)	1.076	0.026	0.53							
Item 3	1.603(.91)	1.362	0.728	0.68	0.50						
Item 4	1.594(.86)	1.461	1.211	0.49	0.64	0.57					
Item 5	1.642(.91)	1.333	0.791	0.52	0.70	0.55	0.77				
Item 6	1.537(.84)	1.439	1.026	0.55	0.50	0.53	0.74	0.73			

Table A.10 (continuation)

Descriptive Statistics and Polychoric Correlations of the Five Scales of the PMDCI

Variables	Mean(*SD*)	Skewness	Kurtosis	Polychoric correlations							
				1	2	3	4	5	6	7	8
Attributions											
Item 1	1.415(.86)	2.021	2.821								
Item 2	1.415(.84)	1.945	2.592	0.90							
Item 3	1.459(.86)	1.843	2.312	0.83	0.82						
Item 4	1.502(.87)	1.641	1.590	0.86	0.85	0.88					
Item 5	1.454(.86)	1.848	2.274	0.92	0.92	0.84	0.90				
Item 6	1.362(.76)	2.257	4.347	0.89	0.86	0.87	0.88	0.90			
Item 7	1.415(.86)	2.103	3.238	0.86	0.89	0.80	0.84	0.94	0.85		
Item 8	1.384(.80)	2.096	3.397	0.90	0.92	0.84	0.89	0.94	0.89	0.92	
Item 9	1.424(.81)	2.013	3.206	0.89	0.90	0.88	0.85	0.91	0.91	0.90	0.93

Table A.11

Proposed UnifactorialModel Parameters of the PMDCI

Scales	Mardia's Coefficient		KMO	Bartlett's Sphericity	Eigenvalues	% Explained Variance	Cronbach's Alpha	McDonalds' Omega	GFI	RMSR
	S	*K*								
Locus Behavior	34.41 < 9(9 + 2) = 99	172.60> 9(9+2) = 99	.86	χ^2_{36} = 1610.7 (p < .001)	5.93	66%	.88	.88 [.84, 91]	.99	0.060
Locus Agency	16.72 < 6(6 + 2) = 48	79.30> 6(6+2) = 48	.84	χ^2_{15} = 1018.4 (p < .001)	4.25	71%	.86	.86 [.80,.89]	.99	0.061
Locus Outcome	26.53 < 6(6 + 2) = 48	112.30 > 6(6 + 2) = 48	.90	χ^2_{15} = 1365.3 (p < .001)	4.73	79%	.89	.89 [85,.92]	1.00	0.046
Locus Recipient	12.58 < 6(6 + 2) = 48	80.21 > 6(6 + 2) = 48	.84	χ^2_{15} = 864.3 (p < .001)	4.01	67%	.83	.83[.77,.87]	.99	0.071
Attributions	96.57<9(9+2) =99	319.51>9(9+2)=99	.95	χ^2_{36} = 2580.8 (p < .001)	8.06	90%	.97	.97 [.95,.97]	1.00	0.020

Table A.12

Exploratory Factor Analysis Parametersand Graded Response Model Parameters for the Five Scales of the PMDCI

Variables	Structure coefficients	Parameters			
		a_1	b_1	b_2	b_3
Locus Behavior scale					
Item 1	0.766	1.191	0.746	1.491	2.232
Item 2	0.870	1.765	0.598	1.289	2.084
Item 3	0.705	0.995	0.163	1.124	2.049
Item 4	0.880	1.852	0.755	1.426	2.128
Item 5	0.754	1.149	0.226	1.263	2.151
Item 6	0.737	1.090	0.462	1.365	1.880
Item 7	0.831	1.493	0.480	1.233	2.057
Item 8	0.758	1.164	0.343	1.212	2.138
Item 9	0.750	1.133	0.516	1.182	1.970
Locus Agency scale					
Item 1	0.689	0.951	0.361	1.172	1.856
Item 2	0.790	1.290	0.659	1.500	2.106
Item 3	0.858	1.667	0.549	1.216	2.051
Item 4	0.788	1.280	0.345	1.166	2.112
Item 5	0.824	1.452	0.499	1.221	2.021
Item 6	0.880	1.853	0.522	1.185	2.060
Locus Outcome scale					
Item 1	0.806	1.363	0.875	1.650	2.121
Item 2	0.795	1.309	0.671	1.464	1.900
Item 3	0.948	2.975	0.446	1.228	1.975
Item 4	0.797	1.321	0.471	1.332	2.034
Item 5	0.915	2.261	0.542	1.204	1.982
Item 6	0.916	2.284	0.528	1.319	1.866
Locus Recipient scale					
Item 1	0.691	0.955	0.199	0.942	2.093
Item 2	0.733	1.078	0.172	1.123	2.015
Item 3	0.710	1.007	0.463	1.343	2.178
Item 4	0.849	1.607	0.333	1.274	1.779
Item 5	0.871	1.771	0.247	1.155	1.696
Item 6	0.798	1.326	0.500	1.283	2.203
Attributions scale					
Item 1	0.936	2.670	0.799	1.220	1.650
Item 2	0.942	2.796	0.765	1.191	1.767
Item 3	0.895	2.009	0.667	1.276	1.768
Item 4	0.920	2.345	0.552	1.155	1.763
Item 5	0.971	4.033	0.643	1.156	1.630
Item 6	0.936	2.662	0.784	1.450	1.826
Item 7	0.932	2.580	0.772	1.320	1.584
Item 8	0.965	3.704	0.760	1.275	1.771
Item 9	0.955	3.236	0.639	1.339	1.698

Appendix A.8

Method selection for Confirmatory Factor Analysis (CFA)

Despite the non-normality of observed data, the procedures that are able to deal with this are rarely used (Yuan & Bentler, 1998). Thus, considering this, we will focus on a few procedures to deal with non-normality that are adequate according to the literature and therefore suitable for the collected data, which is categorical with 4 response categories and non-normally distributed.

We know that the selection of methods in the CFA influences the results that are obtained from the same data, because each estimation method origins different model parameters, standard errors and model data fit values that may change the results (Koğar & Koğar, 2015). Thus, since it can be difficult to know which estimation method may be the most appropriate to model ordered categorical data, we will follow the suggestion of Wirth and Edwards (2007) of fitting the model using multiple estimation methods and then compare results and see if they lead us to equivalent conclusions.

It is necessary to attend to the assumptions of SEM, and then selecting the appropriate method based on the characteristics of the data, because violating the assumptions can lead to data-model fit bias, and consequently, might end in incorrect decisions regarding a given theory that is being tested. Thus, it is essential to remember two main assumptions needed for SEM analysis: metric of the data and its distribution (Finney & DiStefano, 2013). We highlight these two assumptions since data from empirical research is often categorical and data from social sciences do not usually follow multivariate normal distribution (Finney &DiStefano, 2013; Yuan &Bentler, 1998), as our collected data.

In structural equation modeling, the most used estimation method is maximum likelihood (ML) (Li, 2014) and it is the default selection in many software packages (Koğar & Koğar, 2015), such as AMOS. In CFA, the adoption of ML considers that the

observed indicators are according to a continuous and multivariate normal distribution, which is not suitable for ordinal variables (Li, 2016a). This is why it is not advised to use ML methods with ordinal observed variables in general (Li, 2016a; Li, 2016b). ML seems to be biased when variables are non-normally distributed and have few categories (Finney & DiStefano, 2006; Bollen, 1989). That is, underestimation of standard errors and considerable inflation of chi-square statistics makes ML less interesting, especially if data diverge moderately from normality (Li, 2016b; Mîndrilă, 2010). Thus, ML can be used with ordinal data, but only if the scale has at least 5 values, and they are treated as continuous (Schumacker & Beyerlein, 2000). Therefore, results with this method would not be significantly biased if the number of response categories is high and the sample is large (Mîndrilă, 2010). Thus, the greater the non-normality, the greater the impact of ML estimation on results (Finney & DiStefano, 2013). Some authors (e.g., Chou & Bentler, 1995; Muthén & Kaplan, 1985) suggest that when univariate skewness and kurtosis is near of 2 and 7 in absolute values respectively, problems may be encountered, however, there is no cutoff regarding multivariate kurtosis (Finney & DiStefano, 2013). Nonetheless, this method is suited and makes unbiased estimations considering large sample sizes, variables that are normally distributed and continuous (Kline, 2005). Since Maximum Likelihood has been widely used and robust estimators are considered superior, we also used this estimation method with Bollen-Stine and bias-corrected confidence intervals bootstrap (500 replications with 95% CI).

Despite these limitations regarding the use of ML with categorical and non-normal data, several robust variations, as well as procedures have been studied to deal with these data characteristics. Finney and DiStefano (2013) refer that Satorra-Bentler scaling method can be applied under these conditions. Moreover, this type of robust correction to normal theory ML is considered a good strategy for categorical variables,

since they are non-normal (Rhemtulla et al., 2012). Furthermore, this method can be used in small samples, large models, and non-normal data (Satorra & Bentler, 1994).

Other estimation procedures are considered. For instance, Finney and DiStefano (2013) consider that Satorra-Bentler methods should only be used when ordered categories of observed data are 6 or more, however, as can be seen in the work of Jackson et al. (2009) this is a method commonly used. Moreover, Del Rey et al. (2016) have used it in similar research as our own with fewer response categories, thus we will also try this method and compare it to alternative suggestions of other authors. Also, several studies with identical constructs (Casas et al., 2013; Tynes et al., 2010) have used ML estimation methods. In agreement with these studies, Schumacker and Lomax (2010) recommend the use of ML estimation for slight to moderate non-normal interval and ordinal data. However, we believe that considering the ML limitations described above, we prefer to use ML robust procedures, such as Satorra-Bentler. Furthermore, other techniques such as bootstrapping have proven to generate less bias than the default ML estimation when samples were large and non-normality was present (Nevitt & Hancock, 2001). For example, the Bollen-Stine Bootstrapping (Bollen & Stine, 1992) is normally used for continuous, yet non-normal distributed data, or variables that have at least five response categories and whose non-normality derives more from the skewness and kurtosis distribution rather than the discrete nature of the variables (Hancock & Liu, 2012). However, this method has been used recently in cyberbullying research with questionnaires with 4 grade responses (Valdés-Cuervo et al., 2021).

Additionally, consistently with the type of data (i.e. ordinal) some methods can be used, such as weighted least square (WLS), diagonally weighted least squares (DWLS) and unweighted least square (ULS) (Koğar & Koğar, 2015). All these methods calculate the polychoric correlation matrix from the observed categorical

variables (Katsikatsou et al., 2012). Another important remark is the type of distribution. For example, WLS estimation can be used with non-normal interval and ordinal data (Schumacker & Lomax, 2010) however, considering its requirements of a large sample size, it is not recommended (Hoyle, 2012) in this study.

Mîndrilă (2010) found in a comparison between DWLS and ML estimation that DWLS is more accurate than ML when data are ordinal and do not meet normality assumption. For example, Del Rey et al. (2015) used DWLS in cyberbullying research. Moreover, DWLS has robust estimators, such as WLSMV (Diagonally weighted least squares robust estimator) (Muthén et al., 1997) which have presented little bias even when data ordinal with few categories and non-normal (Bandalos & Finney, 2010). Furthermore, Forero et al., 2009 compared DWLS and ULS, and found that the latter outperforms the former in terms of estimation accuracy.

Thus, we selected ULS estimation method because of its consistency, and the fact that it does not makes distributional assumption regarding the observed variables (Bollen, 1989). Moreover, Jöreskog and Sörbom (1989) defend that when Likert scales are used, it is advisable to use polychoric correlations, and therefore estimation methods, such as ULS, are preferred. Likewise, ULS outperforms DWLS in terms of estimation accuracy (Forero et al., 2009) and has also better results in lower samples than DWLS (Koğar & Koğar, 2015).

Appendix A.9

Fit Indices

Several fit indices will be presented according to the ones available for each estimation procedure. Thus, in order to arrange all fit indices in a logical manner in the following tables (for CFA regarding EQVC and PMDCI), they will be presented following their main classification. That is, first Absolute fit indices (Byrne, 2010;

Marôco, 2014) or Residual-based fit indices (Jöreskog & Sörbom, 1981), such as $\chi 2/df$,Goodness of Fit Index (GFI) and Adjusted Goodness of Fit Index (AGFI) (Byrne, 2010; Marôco, 2014) and Root Mean Square Residual (RMSR) (Marôco, 2014; Jöreskog & Sörbom, 1981). Then, Comparative or Incremental Fit indices (Marôco, 2014; Byrne, 2010) such as Comparative Fit Index (CFI), Normed Fit Index (NFI), Tucker-Lewis Index (TLI), Incremental Fit Index (IFI), followed by Parsimony fit indices such as Parsimony Goodness of Fit Index (PGFI) and Parsimony Normed Fit Index (PNFI). Lastly, we present Fit Indices for Comparing Non-nested Models (such as Root Mean Square of Approximation (RMSEA), also categorized by others as an Absolute Fit Index, or as Population Index (Marôco, 2014) and Akaike's Information Criterion (AIC), also described by others (Hayashi et al., 2011; Marôco, 2014) as Information-criterion-based fit index.

In order to assess the model fit, the following indexes and values were taking into consideration. GFI is an absolute fit index with a corresponding adjusted version, the AGFI (Jöreskog & Sörbom, 1984). Both GFI and AGFI have values between 0 and 1, with 1 indicating a perfect fit. As suggested by Cole (1987), a value of .80 for the AGFI has usually been considered as the minimum for model acceptance. Moreover, parsimony based fit measures larger than .60 are generally considered satisfactory (Blunch, 2008).The other estimation methods also include the following fit indices: CFI and IFI values close to 1 indicate a good statistical fit (Bentler, 1990), TLI values >.90 indicates acceptable fit (Bentler & Bonett, 1980), while RMSEA indicates a good fit if equal or less than .08 (Browne & Cudeck, 1993). As for the AIC, the lower the value, the better the fit of the model. Finally, the SRMR should be close to zero for a good fit.

Table A.13

Fit Index Values for the Three Models Tested and the Four Estimation Methods of the CFA of the EQVC

Estimation method	CFA Models	χ^2	*df*	Sig.	χ^2/df	GFI	AGFI	SRMR	CFI	IFI	NFI	TLI	PGFI	PNFI	RMSEA	AIC
ULS	Model 1	168.881	151	-	1.118	.966	.957	.056	-	-	.924	-	.768	.816	-	-
	Model 2	169.216	151	-	1.121	966	,957	.056	-	-	.925	-	,768	.816	-	-
	Model 3	157.528	149	-	1.057	.968	.960	.054	-	-	.930	-	.759	.810	-	-
WLSMV	Model 1	230.474	151	.000	1.526	.978	.972	.056	.923	.924	.808	.913	.777	.815	.039	-
	Model 2	240.096	151	.000	1.590	.977	.971	.057	.915	.916	.802	.903	.776	.814	.042	-
	Model 3	228.526	149	.000	1.534	.978	.972	.055	.924	.925	.812	.913	.767	.807	.040	-
ML Satorra-Bentler correction	Model 1	248.345	151	.000	1.645	.916	.895	.057	.897	.899	.777	.883	.728	.682	.043	17071.630
	Model 2	253.922	151	.000	1.682	.914	.892	.055	.892	.895	.776	.877	.727	.683	.045	16900.742
	Model 3	234.831	149	.000	1.576	.921	.900	.057	.911	.913	.793	.898	.723	.689	.041	16881.181
ML Bollen-Stine Bootstrapping	Model 1	295.487	151	.000	1.957	.916	.895	.057	.872	.874	.772	.855	.728	.682	.053	373.487
	Model 2	297.053	151	.000	1.967	.915	.893	.057	.872	.874	.773	.855	.727	.683	.053	375.053
	Model 3	270.940	149	.000	1.818	.923	.902	.055	.893	.895	.793	.886	.724	.691	.049	352.940

Note: Instead of χ^2 is presented SB- χ^2 for all ML with Satorra-Bentler correction, Scaling correction factor for Model 1= 1.193, Model 2= 1.176 and Model 3=1.172. ML Bollen-Stine Bootstrapping – Model 1: *p* = .002, RMSEA [.044, .062]; Model 2: *p* = .005, RMSEA [.042, .060]; Model: 3: *p* = .002, RMSEA [.038, .056].

Figure A.1

Diagram from the CFA for the two-factor model of the EQVC (with standardized factor loadings)

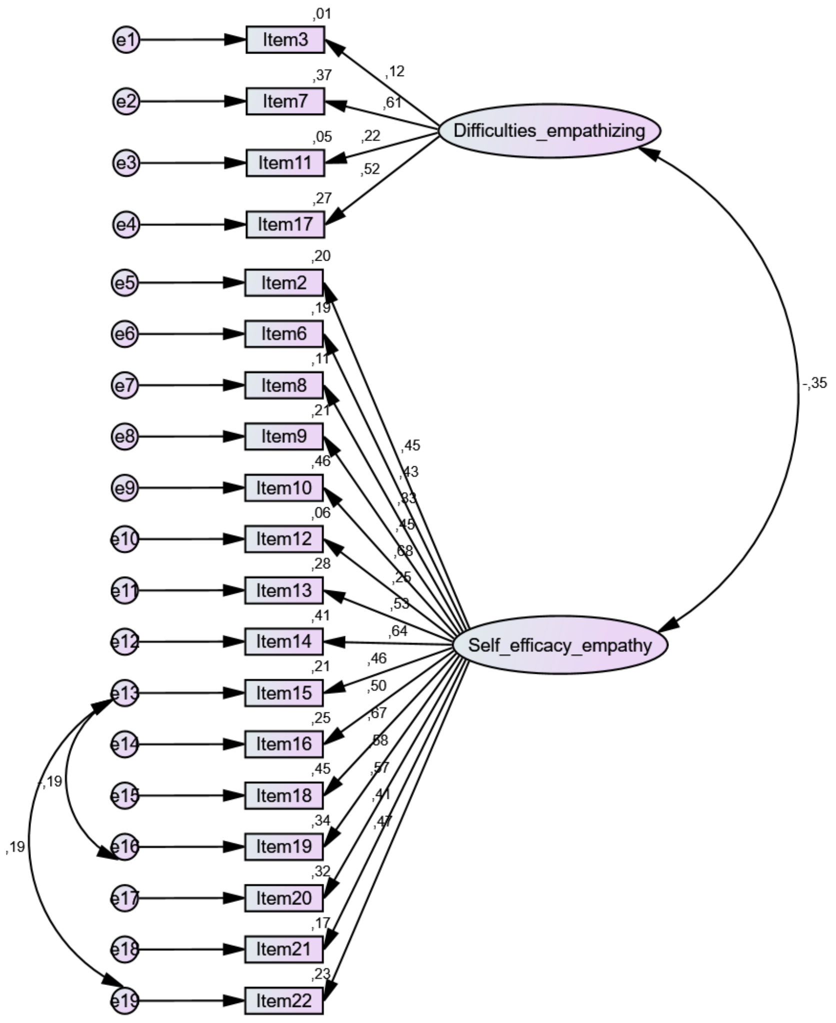

Note. For model 3 we established covariances between the error terms of the items with the similar processes (i.e., item 15 – "My online friends tell me about their problems because they say I am very understanding" and item 19 – "I can easily understand what someone else wants to talk about online", and item 15 and item 22 – "I easily get emotionally involved in my online friends' problems").

Table A.14

Fit Index Values for the Three Models Tested and the Four Estimation Methods of the CFA of the Locus Behavior Scale of the PMDCI

Estimation method	CFA Models	χ2	*df*	Sig.	χ2/*df*	GFI	AGFI	SRMR	CFI	IFI	NFI	TLI	PGFI	PNFI	RMSEA	AIC
ULS	Model 1	30.074	27	-	1.114	.973	.956	.074	-	-	.931	-	.584	.698	-	-
	Model 2	27.330	27	-	1.012	.974	.956	.071	-	-	.930	-	.584	.698	-	-
	Model 3	9.638	25	-	.386	.991	.983	.050	-	-	.975	-	.550	.677	-	-
WLSMV	Model 1	71.738	27	.000	2.657	.996	.992	.068	.881	.884	.826	.842	.498	.690	.070	-
	Model 2	77.638	27	.000	2.875	.996	.993	.064	.870	.873	.818	.827	.498	.691	.074	-
	Model 3	39.534	25	.033	1.581	.998	.996	.045	.963	.964	.907	.946	.462	.669	.041	-
ML Satorra-Bentler correction	Model 1	90.556	27	.000	3.354	.975	.950	.065	.832	.836	.781	.776	.487	.575	.107	6702.097
	Model 2	90.059	27	.000	3.336	.979	.957	.063	.841	.844	.792	.788	.489	.587	.101	6500.132
	Model 3	44.020	25	.011	1.761	.990	.978	.045	.951	.953	.898	.929	.458	.622	.057	6435.202
ML Bollen-Stine Bootstrapping	Model 1	148.919	27	.000	5.516	.911	.852	.072	.797	.800	.766	.730	.547	.575	.115	184.919
	Model 2	131.812	27	.000	4.882	.920	.866	.069	.816	.819	.783	.755	.552	.587	.107	167.812
	Model 3	63.085	25	.000	2.523	.960	.929	.049	.933	.934	.896	.904	.533	.622	.067	103.085

Note: 3 outliers were removed in Model 2. Instead of χ^2 is presented SB- χ^2 for all ML with Satorra-Bentler correction, Scaling correction factor for Model 1= 1.649, Model 2= 1.468 and Model 3=1.437. ML Bollen-Stine Bootstrapping – Model 1: p = .002, RMSEA [.097, .133]; Model 2: p = .002, RMSEA [.089, .126]; Model: 3 p = .018, RMSEA [.047, .088].

Figure A.2

Diagram from the CFA for the Locus of Behavior scale (with standardized factor loadings)

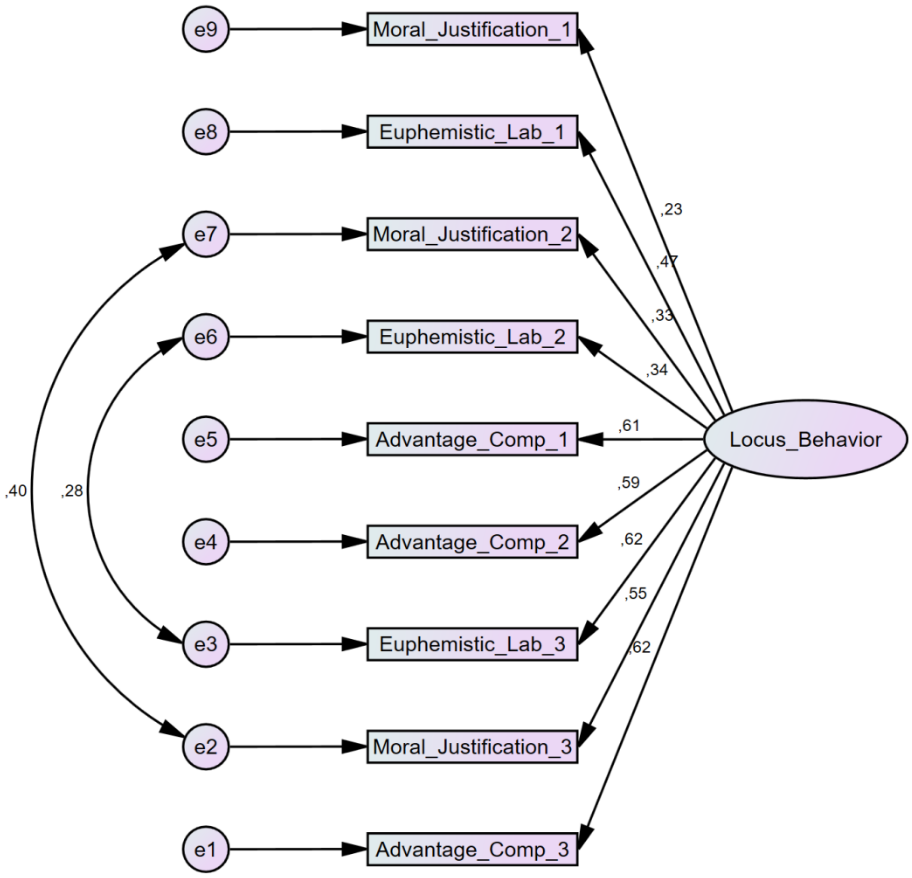

Note. Model 3has the covariances between the error terms of items “It’s okay if I see someone insulting someone else online who mistreated his/her family.” And “It doesn’t matter if I see someone making fun of someone else online who mistreated his/her friends.”, and between items “If I see someone sexually harassing someone else online, I think it’s just a joke.” and “If I see someone making fun of someone else online, I think he/she is just kidding.”

Table A.15

Fit Index Values for the Three Models Tested and the Four Estimation Methods of the CFA of the Locus Agency Scale of the PMDCI

Estimation method	CFA Models	$\chi 2$	*df*	Sig.	$\chi 2/df$	GFI	AGFI	SRMR	CFI	IFI	NFI	TLI	PGFI	PNFI	RMSEA	AIC
ULS	Model 1	3.853	9	-	.428	.991	.980	.051	-	-	.965	-	.425	.579	-	-
	Model 2	4.344	9	-	.483	.989	.975	.058	-	-	.955	-	.424	.573	-	-
	Model 3	1.233	8	-	.154	.997	.992	.032	-	-	.987	-	.380	.526	-	-
WLSMV	Model 1	11.921	9	.218	1.325	.999	.997	.044	.974	.976	.907	.957	.333	.572	.031	-
	Model 2	16.723	9	.053	1.858	.999	.996	.051	.941	.943	.885	.901	.333	.560	.050	-
	Model 3	8.538	8	.383	1.067	.999	.998	.027	.996	.996	.941	.992	.296	.516	.014	-
ML Satorra-Bentler correction	Model 1	12.981	9	.163	1.442	.995	.984	.044	.964	.967	.901	.940	.332	.537	.051	4244.105
	Model 2	19.447	9	.022	2.161	.994	,981	.050	.915	.926	.870	.859	.331	.515	.078	4083.202
	Model 3	7.568	8	.477	.946	.998	.993	.027	1.00	1.003	.950	1.006	.296	.507	.000	4062.705
ML Bollen-Stine Bootstrapping	Model 1	26.053	9	.002	2.895	.975	.941	.050	.927	.929	.896	.879	.418	.537	.075	50.053
	Model 2	34.467	9	.000	3.830	.968	.924	.057	.888	.891	.858	.814	.415	.515	.091	58.467
	Model 3	12.037	8	.150	1.505	.989	.970	.031	.982	.983	.950	.967	.377	.507	.039	38.037

Note: 3 outliers were removed in Model 2. Instead of χ^2 is presented SB- χ^2 for all ML with Satorra-Bentler correction, Scaling correction factor for Model 1=2.013, Model 2=1.626 and Model 3=1.595. ML Bollen-Stine Bootstrapping – Model 1: *p* = .186, RMSEA [.042, .109]; Model 2: *p*= .034, RMSEA [.060, .125]; Model: 3 *p*= .439, RMSEA [.000, .081].

Figure A.3

Diagram from the CFA for the Locus of Agency scale (with standardized factor loadings)

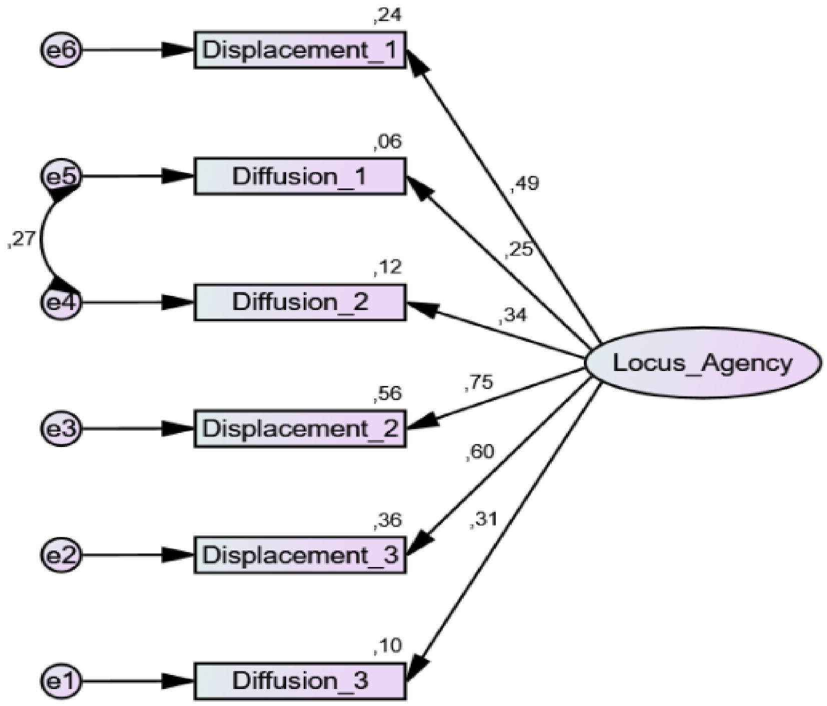

Note. Model 3 has the covariances between two error terms of items "If I see someone sexually harassing someone else online, I think that that person should not be held responsible because everyone in the group does the same." and "If I see someone insulting someone else online, I think that that person should not be held responsible because he/she is not the only one doing so."

Table A.16

Fit Index Values for the Three Models Tested and the Four Estimation Methods of the CFA of the Locus Outcome Scale of the PMDCI

Estimation method	CFA Models	χ2	*df*	Sig.	χ2/*df*	GFI	AGFI	SRMR	CFI	IFI	NFI	TLI	PGFI	PNFI	RMSEA	AIC
ULS	Model 1	.691	9	-	.077	.998	.995	.024	-	-	.995	-	.428	.597	-	-
	Model 2	.904	9	-	.100	.997	.993	.028	-	-	.993	-	.427	.596	-	-
WLSMV	Model 1	7.160	9	.620	.796	1.00	.999	.021	1.00	1.011	.961	1.018	.333	.596	.00	-
	Model 2	9.523	9	.390	1.058	1.00	.999	.025	.997	.997	.949	.995	.333	.594	.013	-
ML Satorra-Bentler correction	Model 1	7.274	9	.609	.808	.998	.993	.021	1.00	1.007	.971	1.008	.333	.588	.00	3518.127
	Model 2	8.943	9	.443	.994	.997	.991	.025	1.00	1.000	.968	1.00	.332	.585	.00	3317.206
ML Bollen-Stine Bootstrapping	Model 1	11.289	9	.256	1.254	.990	.976	.024	.996	.996	.980	.993	.424	.588	.027	35.289
	Model 2	15.427	9	.080	1.714	.986	.968	.028	.990	.990	.976	.983	.423	.585	.046	39.427

Note: 4 outliers were removed in Model 2. Instead of χ^2 is presented SB- χ^2 for all ML with Satorra-Bentler correction, Scaling correction factor for Model 1=1.557, Model 2=1.730. ML Bollen-Stine Bootstrapping – Model 1: p = .613, RMSEA [.000, .070]; Model 2: p = .419, RMSEA [.000, .084].

Figure A.4

Diagram from the CFA for the Locus of Outcome scale (with standardized factor loadings)

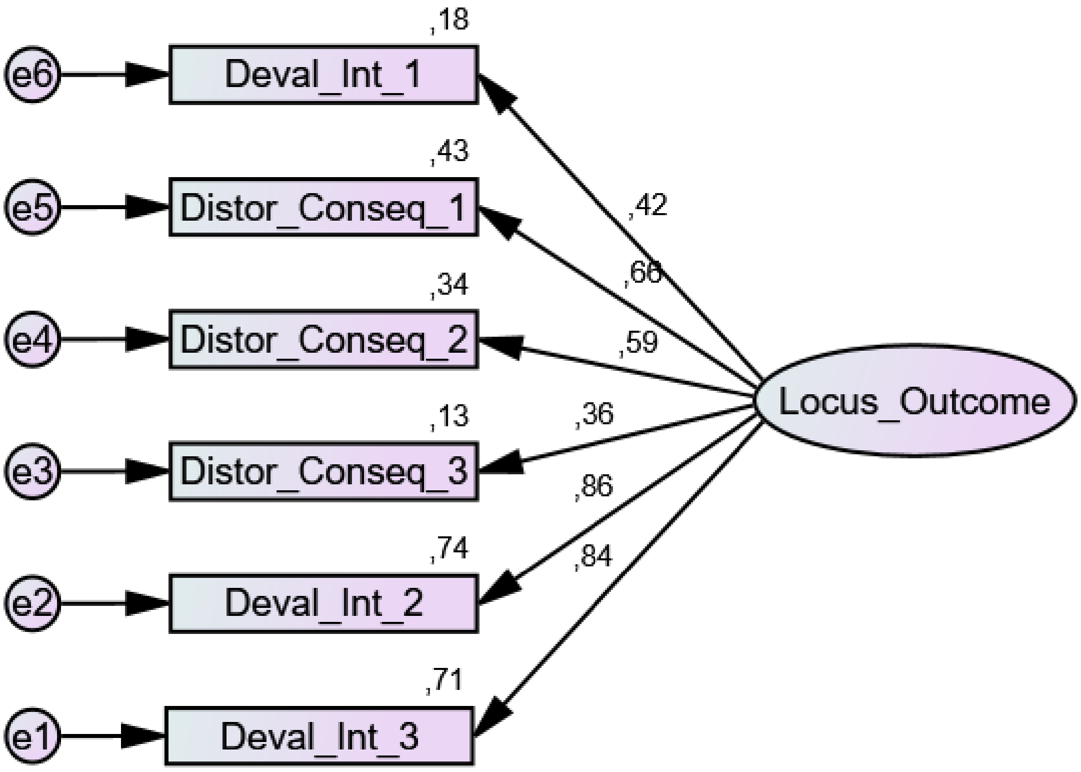

Table A.17

Fit Index Values for the Three Models Tested and the Four Estimation Methods of the CFA of the Locus Recipient Scale of the PMDCI

Estimation method	CFA Models	χ2	*df*	Sig.	χ2/*df*	GFI	AGFI	SRMR	CFI	IFI	NFI	TLI	PGFI	PNFI	RMSEA	AIC
ULS	Model 1	57.142	9	-	6.349	.939	.858	.104	-	-	.810	-	.402	.486	-	-
	Model 2	59.483	9	-	6.609	.935	.848	.108	-	-	.800	-	.401	.480	-	-
	Model 3	6.366	7	-	.909	.993	.979	.042	-	-	.979	-	.331	.457	-	-
WLSMV	Model 1	100.445	9	.000	11.161	.993	.980	.092	.524	.539	.515	.207	.331	.491	.173	-
	Model 2	108.564	9	.000	12.063	.993	.979	.096	.479	.495	.473	.132	.331	.488	.181	-
	Model 3	19.890	7	.006	2.841	.999	.996	.037	.933	.935	.903	.855	.259	.455	.074	-
ML Satorra-Bentler correction	Model 1	102.978	9	.000	11.442	.975	.924	.097	.733	.682	.661	.554	.325	.433	.192	4965.521
	Model 2	113.887	9	.000	12.654	.972	.917	.100	.714	.656	.637	.523	.324	.422	.204	4872.549
	Model 3	20.857	7	.004	2.980	.994	.978	.041	.962	.955	.934	.919	.258	.441	.084	4764.542
ML Bollen-Stine Bootstrapping	Model 1	123.462	9	.000	13.718	.892	.748	.110	.733	.736	.722	.555	.382	.433	.193	147.462
	Model 2	136.858	9	.000	15.206	.884	.729	.113	.714	.718	.704	.523	.379	.422	.205	160.858
	Model 3	25.182	7	.001	3.597	.976	.928	.046	.959	.960	.945	.913	.325	.441	.088	53.182

Note: 3 outliers were removed in Model 2. Instead of χ^2 is presented SB- χ^2 for all ML with Satorra-Bentler correction, Scaling correction factor for Model 1=1.202, Model 2=1.205 and Model 3=1.211. ML Bollen-Stine Bootstrapping – Model 1: p = .002, RMSEA [.164, .224]; Model 2: p = .002, RMSEA [.175, .236]; Model 3: p = .004, RMSEA [.052, .126].

Figure A.5

Diagram from the CFA for the Locus of Recipient scale (with standardized factor loadings)

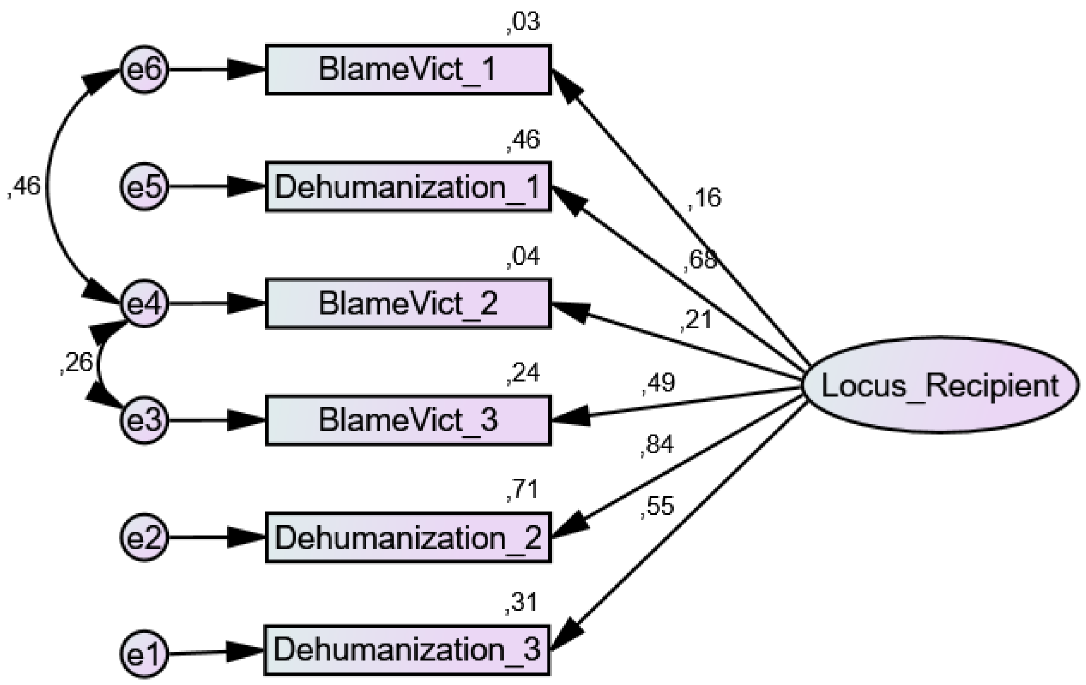

Note. Model 3 has the covariances between the error terms of items "If I see someone sexually harassing someone else online, it is because that person showed too much in the photos he/she posted" and "If I see someone making fun of someone else online, it is because that person overexposed him/herself.", and between items "If I see someone making fun of someone else online, it is because that person overexposed him/herself" and "If I see someone insulting someone else online, it is because that person deserved it."

Table A.18

Fit Index Values for the Three Models Tested and the Four Estimation Methods of the CFA of the Attributions Scale of the PMDCI

Estimation method	CFA Models	χ2	*df*	Sig.	χ2/*df*	GFI	AGFI	SRMR	CFI	IFI	NFI	TLI	PGFI	PNFI	RMSEA	AIC
ULS	Model 1	2.167	27	-	.080	.991	.985	.053	-	-	.985	-	.595	.739	-	-
	Model 2	1.505	27	-	.056	.990	.984	.056	-	-	.984	-	.594	.738	-	-
	Model 3	1.198	26	-	.046	.992	.987	.050	-	-	.987	-	.573	.713	-	-
WLSMV	Model 1	29.324	27	.345	1.086	1.000	.999	.049	.961	.966	.692	.948	.500	.738	.016	-
	Model 2	31.246	27	.261	1.157	1.000	1.000	.052	.917	.929	.641	.889	.500	.738	.022	-
	Model 3	30.390	26	.252	1.169	1.000	1.000	.052	.914	.928	.651	.881	.481	.711	.023	-
ML Satorra-Bentler correction	Model 1	29.865	27	.320	1.106	.973	.947	.048	.986	.974	.786	.982	.487	.671	.039	2918.870
	Model 2	37.377	27	.088	1.384	.977	.953	.051	.954	.899	.712	.938	.488	.660	.068	2403.319
	Model 3	36.942	26	.076	1.421	.976	.951	.051	.951	.894	.715	.932	.470	.636	.071	2403.797
ML Bollen-Stine Bootstrapping	Model 1	142.241	27	.000	5.268	.907	.846	.053	.912	.913	.895	.883	.544	.671	.114	178.241
	Model 2	146.591	27	.000	5.429	.902	.837	.056	.899	.899	.879	.865	.541	.660	.116	182.591
	Model 3	115.388	26	.000	5.580	.927	.874	.050	.924	.925	.905	.895	.536	.654	.103	153.388

Note: 4 outliers were removed in Model 2. Instead of χ^2 is presented SB- χ^2 for all ML with Satorra-Bentler correction, Scaling correction factor for Model 1=.4777, Model 2=3.934 and Model 3=3.939. ML Bollen-Stine Bootstrapping – Model 1: p = .218, RMSEA [.096, .132]; Model 2: p = .142, RMSEA [.098, .135]; Model 3: p = .148, RMSEA [.084, .122].

Figure A.6

Diagram from the CFA for the Attributions scale (with standardized factor loadings)

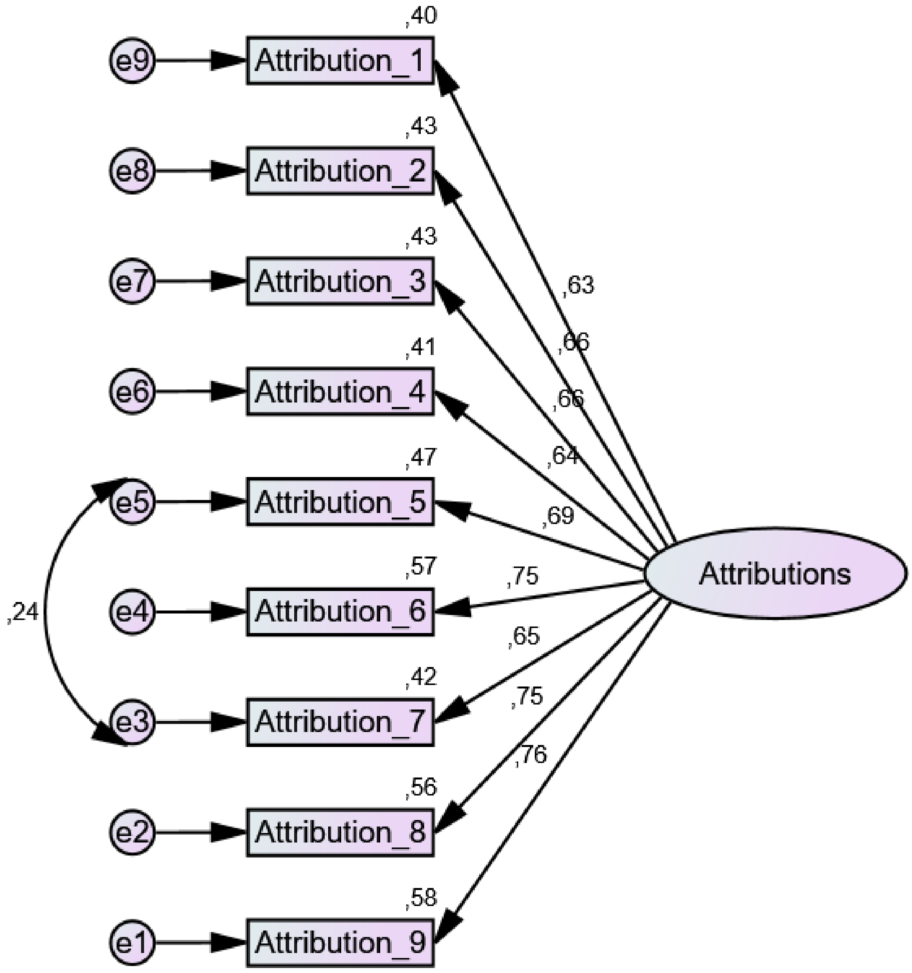

Note. Model 3 has the covariances between two error terms of items “It doesn’t matter if I see someone sexually harassing someone else online because he/she doesn’t like that person” and “It doesn’t matter if I see someone sexually harassing someone else online because he/she wants to be part of an online group where that is normal”.

Appendix A.10

With respect to the EQVC, the factor Difficulties in Empathizing refers to difficulties that adolescents tend to feel when interacting online with their friends, particularly with respect to empathize with them. Moreover, the factor Self-efficacy regarding empathy refers to a feeling from adolescents, of being able to empathize with their friends in the online context.

With respect to the PMDCI, it is composed of 4 moral disengagement scales, each comprising a different locus, and an Attribution scale. The Behavior scale includes moral disengagement mechanisms of Moral Justification, Euphemistic labeling and Advantage Comparison, and refers to how adolescents use cognitive reconstruction to justify the detrimental conduct. The Agency scale includes moral disengagement mechanisms of Displacement of responsibility and Diffusion of responsibility, and it operates within the agentic role in the harmful conduct. The Outcome scale includes moral disengagement mechanisms of Devaluation of behavioral intentions and Distortion of consequences, which allows insidious acts to be ignored in some and self-censure is mostly likely not activated (Bandura, 2002). And finally, the Recipient scale includes moral disengagement mechanisms of Attribution of blame and Dehumanization, which tend to decrease self-censure, considering how the victim is regarded. With respect to the Attribution scale, it includes Absence of affective bonding, Aggressors' factors and Interpersonal relationships, and this scale reflects motives for engaging in cyberbullying.
